# Haplotype-specific *MAPT* exon 3 expression regulated by common intronic polymorphisms associated with Parkinsonian disorders

**DOI:** 10.1186/s13024-017-0224-6

**Published:** 2017-10-30

**Authors:** Mang Ching Lai, Anne-Laure Bechy, Franziska Denk, Emma Collins, Maria Gavriliouk, Judith B. Zaugg, Brent J. Ryan, Richard Wade-Martins, Tara M. Caffrey

**Affiliations:** 1 Department of Physiology, Anatomy and Genetics, University of Oxford, Oxford, OX1 3QX UK; 2 European Molecular Biology Laboratory, 69117 Heidelberg, Germany; 3 Oxford Parkinson’s Disease Centre, University of Oxford, Oxford, OX1 3QX UK

**Keywords:** Tau, Progressive supranuclear palsy, Alzheimer’s disease, *MAPT*, Corticobasal degeneration, Parkinson’s disease

## Abstract

**Background:**

Genome wide association studies have identified microtubule associated protein tau (*MAPT*) H1 haplotype single nucleotide polymorphisms (SNPs) as leading common risk variants for Parkinson’s disease, progressive supranuclear palsy and corticobasal degeneration. The *MAPT* risk variants fall within a large 1.8 Mb region of high linkage disequilibrium, making it difficult to discern the functionally important risk variants. Here, we leverage the strong haplotype-specific expression of *MAPT* exon 3 to investigate the functionality of SNPs that fall within this H1 haplotype region of linkage disequilibrium.

**Methods:**

In this study, we dissect the molecular mechanisms by which haplotype-specific SNPs confer allele-specific effects on the alternative splicing of *MAPT* exon 3. Firstly, we use haplotype-hybrid whole-locus genomic *MAPT* vectors studies to identify functional SNPs. Next, we characterise the RNA-protein interactions at two loci by mass spectrometry. Lastly, we knockdown candidate splice factors to determine their effect on *MAPT* exon 3 using a novel allele-specific qPCR assay.

**Results:**

Using whole-locus genomic DNA expression vectors to express *MAPT* haplotype variants, we demonstrate that rs17651213 regulates exon 3 inclusion in a haplotype-specific manner. We further investigated the functionality of this region using RNA-electrophoretic mobility shift assays to show differential RNA-protein complex formation at the H1 and H2 sequence variants of SNP rs17651213 and rs1800547 and subsequently identified candidate trans-acting splicing factors interacting with these functional SNPs sequences by RNA-protein pull-down experiment and mass spectrometry. Finally, gene knockdown of candidate splice factors identified by mass spectrometry demonstrate a role for hnRNP F and hnRNP Q in the haplotype-specific regulation of exon 3 inclusion.

**Conclusions:**

We identified common splice factors hnRNP F and hnRNP Q regulating the haplotype-specific splicing of *MAPT* exon 3 through intronic variants rs1800547 and rs17651213. This work demonstrates an integrated approach to characterise the functionality of risk variants in large regions of linkage disequilibrium.

**Electronic supplementary material:**

The online version of this article (10.1186/s13024-017-0224-6) contains supplementary material, which is available to authorized users.

## Background

Genome wide association studies (GWAS) provide a powerful tool for identifying common genetic variation associated with complex common traits. Single nucleotide polymorphisms (SNPs) represent the most common form of variation in the human genome [1]. Most genotyping platforms used in GWAS use approximately 1 million SNPs to capture this genomic diversity [2]. As the SNPs sampled in GWAS account for less than 10% of all SNPs present in the genome, the causative SNPs are unlikely to be sampled themselves and are thus more likely to be found in linkage disequilibrium (LD) with the GWAS risk SNPs [3]. As protein-coding regions make up only about 1% of the ∼3.3 billion nucleotides in the human genome [4], it is not surprising that the majority of GWAS risk SNPs identified map to non-coding sequences [5–8]. Together with the recent advancements in post-GWAS interpretation methods, increasing evidence has pointed towards the enrichment and the functionality of GWAS risk variants and their associated SNPs in non-coding regulatory elements such as epigenetic markers, transcription factor binding sites, DNase I hypersensitive sites, RNA splicing and gene expression [7, 9–12]. All of the above highlight the importance of understanding the functional polymorphisms within large expanses of LD which is often challenging due to the difficulty of working with large genomic regions in models of disease and the potential subtle effects of functional polymorphisms.

The microtubule-associated protein tau (*MAPT*) locus is among the most important gene loci in neurodegeneration implicated in genetic risk for or pathology of a number of neurodegenerative disorders. There are two principal genetic haplotypes at the locus, named H1 and H2, of which the H1 haplotype shows strong genetic association with a number of neurodegenerative diseases including progressive supranuclear palsy (PSP) (odds-ratio [OR] of 5.5) [13], corticobasal degeneration (CBD) (OR 3.7) [14] and Parkinson’s disease (PD) (OR 1.3) [15, 16]. Linkage disequilibrium across the region is very high (~1.8 Mb) due to the presence of a 900 kb chromosomal inversion on the H2 haplotype [17], making it particularly challenging to identify functionally important polymorphisms. Prior to *MAPT* being identified in genetic association studies as a risk locus for PD, PSP and CBD, the tau protein was already of interest in a number of neurodegenerative disorders due to the presence of abnormally phosphorylated tau protein in pathological aggregations in the form of neurofibrillary tangles. The multiple biological involvements of tau in neurodegeneration places further importance of understanding tau biology at both the genetic and the protein levels.

Our laboratory is interested in the hypothesis that polymorphisms within the *MAPT* sequence have functional consequences on *MAPT* gene splicing and therefore protein function [18–20]. Mutations at the exon 10 splice site in FTDP-17 patients demonstrate that differences in splicing alone are sufficient to generate disease [21, 22]. We have previously shown the H1 haplotype expresses up to 40% greater exon 10 containing transcripts than H2 in the absence of overall transcript expression differences [18]. Additionally, we have shown that the H2 haplotype expresses 2-fold greater transcripts containing the alternatively spliced exon 3, both in cells, post-mortem brain tissue [19] and induced pluripotent stem cell derived models of neurodegeneration [23]. Recently, 2N tau isoforms have been show to interact with proteins important for neurodegenerative pathways (Parkinson’s, Alzheimer’s and Huntington’s disease) [24]. Additionally, there is evidence that 2N isoforms depress tau aggregation [25] which together may indicate 2N tau proteins offer some protection from pathology. *MAPT.*

Here, we present an approach to determine the functional effects of specific SNPs located within a large region of LD*MAPT* by leveraging the strong haplotype specific expression of *MAPT* exon 3 to gauge SNP functionality. Our laboratory uses high capacity bacterial and P1-phage artificial chromosome vectors (BACs and PACs) to express whole genomic loci in culture and applies homologous recombination technology to manipulate the large inserts with base-pair accuracy. We have previously used these vector systems to express the human *MAPT* locus in neuronal cell culture models and demonstrated *MAPT* locus expression is under developmental and cell-type specific regulation [26] and in transgenic mouse models express all six adult tau isoforms [27]. Here, we applied an analogous strategy to generate genomic DNA p*MAPT*-H1 and p*MAPT*-H2 expression vectors with identical upstream and downstream sequence, differing only at sites of haplotype variation. We generated haplotype hybrid vectors using homologous recombination in *E. coli* to specifically assay the impact of polymorphisms on splice phenotypes observed at *MAPT* exon 3. To understand the underlying mechanisms of the haplotype-specific splicing regulation, we employed biochemical techniques to study the impact of H1/H2 SNP sequences on RNA-protein interaction and to identify their interacting trans-acting splicing regulators. We developed an allele-specific qPCR assay to measure the ratios of H1 vs H2 *MAPT* transcripts in our RNA interference experiments and identified hnRNP F and hnRNP Q as critical protein regulators of haplotype-specific inclusion exon 3.

## Methods

### Generation of p*MAPT* hybrid vectors

Homologous recombination technology from GeneBridges for BAC modifications using a selection-counter selection method [28] was used for engineering the p*MAPT* vectors. Briefly, PCR products was produced using primers that amplify the selection-counter selection streptomycin sensitive/chloramphenicol resistant (rpsl/chl) cassette, with long homology arms flanking the p*MAPT* sequence to be modified. The PCR product containing the rpsl/chl cassette was then used to replace the p*MAPT* sequence to be modified by homologous recombination. Bacterial colonies were selected based on streptomycin sensitivity and chloramphenicol resistance. The rpsl/chl cassette was then excised and replaced by PCR products containing the desired sequence using homologous recombination. Colonies were selected on streptomycin resistance and colony PCR was performed to screen for the deletion of the rpsl/chl cassette and the insertions of the engineered sequence. DNA sequencing was performed to identify bacterial colonies with sequence successfully modified. Primer sequences are given in Additional file 1: Table S1.

### Transfection of SK-N-F1 Neuroblastoma cells

SK-N-F1 Neuroblastoma cells were cultured in culturing medium [Dulbecco’s modified eagle’s medium (Sigma-Aldrich) with 10% fetal bovine serum, 4 mM L-glutamine, 50 U/mL penicillin and 50 μg/mL streptomycin, and 1X minimum essential medium non-essential amino acids (Life Technologies)]. 7–10 × 10^5^ cells were seeded per 6-well well. Transfection was carried out when cell confluence reached ~75%. 6.25 μg of each *MAPT* construct DNA was incubated for 10 min at room temperature with 6.25 μL of PLUS™ reagent in Opti-MEM® reduced serum medium (Life Technologies). 15.6 μL of Lipofetamine® LTX reagent (Life Technologies) diluted in Opti-MEM® medium and then incubated with the DNA mixture for a further 30 min, forming the transfection mix. Cells were washed with Opti-MEM® medium and were then incubated with the transfection mix for 4 h at 37 °C, 5% CO_2_. Cells were washed with OptiMEM® medium and were then further incubated in culturing medium for 48 h before proceeding to RNA extraction.

### RNA extraction and cDNA synthesis

SK-N-F1 cells were harvested in TRIzol reagent (Life Technologies). Organic and aqueous phases were separated by the addition of chloroform. Total RNA was precipitated by the addition of isopropanol. RNA purification was then carried out using the RNeasy mini kit (Qiagen) according to the manufacturer’s protocol. RNA concentration was determined using the ND-1000 nanodrop spectrophotometer. cDNA was generated using either SuperScript® III Reverse Transcriptase (Life Technologies) or SuperScript® VILO Master Mix (Life Technologies) from 1 to 5 μg RNA according to the manufacturer’s protocol.

### qPCR

Quantitative PCR was performed using either SYBR® Green PCR Master Mix or Fast SYBR® Green PCR Master Mix and StepOnePlus™ System (Life Technologies) according to manufacturer’s protocol and cycling parameters. For measuring transgenic total *MAPT* and exon 3 expression, a relative standard curve method was employed to compare the amount of exon 3 transcripts expressed to that of total *MAPT* and values obtained are in arbitrary units for internal comparisons not a percentage of the total tau expression. For measuring splice factor expression levels, a 2^-(delta-delta Ct)^ method [29] was used where gene expression is normalised against the geometric means of three endogenous control genes *GAPDH*, *HPRT1* and *ACTB* [30]. All qPCR reactions were performed in triplicate. Primer sequences (Eurofins Scientific and Integrated DNA Technologies) are listed in Additional file 1: Table S2.

### Allele-specific qPCR assay

H1 (FAM labelled) and H2 (VIC labelled) specific Taqman probes spanning rs17650901 in exon 1 were designed using Primer Express 3.0 (Applied Biosystems) and Primer 3 [31, 32]. A standard curve was generated using genomic primers where the delta Ct values between FAM (H1) and VIC FAM (H2) signals were plotted against the log_2_ ratios of H1:H2 transcripts by mixing 50 pg of H1 and H2 p*MAPT* vectors in the ratios 8:1, 4:1, 2:1, 1:1, 1:2, 1:4 and 1:8. The equation of regression line obtained from the standard curve log_2_ (H1/H2) = −1.194 × delta Ct was used calculate the ratios of H1:H2 transcripts. Primers (Integrated DNA Technologies) and probes (Life Technologies) are listed in Additional file 1: Table S3.

### RNA Electrophoretic mobility shift assay (EMSA)

SK-N-F1 nuclear lysate was extracted using a protocol as previously described [33] with minor modifications. Briefly, SK-N-F1 cells were grown in 15 cm dishes for 48 h and were harvested by gentle scraping. The cytoplasmic fraction was first extracted using cold lysis buffer (10 mM HEPES, 10 mM KCl, 0.1 mM EDTA, 0.1 mM EGTA, Halt protease and phosphatase inhibitors cocktail and 0.67% Igepal) (Ambion, Thermo Scientific and Sigma Aldrich). The nuclear pellet was washed and lysed using cold nuclear lysis buffer (20 mM HEPES, 400 mM KCl, 1 mM EDTA, 1 mM EGTA and Halt protease and phosphatase inhibitors cocktail) (Ambion, Thermo Scientific and Sigma Aldrich). RNA EMSA was carried out using RNA oligonucleotides biotinylated at the 3′ end and SK-N-F1 nuclear lysates using the LightShift Chemiluminescent RNA EMSA Kit (Pierce) according the manufacturer’s protocol. rs17651213 H1 (5’TGA GGG AGC TTT GCG TGT TTA TCC TCC TGT3’) and H2 (5’TGA GGG AGC TTT GCA TGT TTA TCC TCC TGT3’) probes were biotinylated at the 3′ end via a 15 atoms triethyleneglycol linker (Integrated DNA Technologies). rs1800547 H1 (5’CCA CAG GUG AGG GUA AGC CCC AGA GAC CCC5’) and H2 (5’CCA CAG GUG AGG GUG AGC CCC AGA GAC CCC3’) probes (Integrated DNA Technologies) were biotinylated at the 3′ end joined by a cytidine (bis)phosphate residue using the RNA 3′ End Biotinylation Kit (Pierce) according to manufacturer’s protocol.

### RNA pull-down

RNA pull-down of RNA binding proteins was performed using the magnetic RNA-protein pull-down kit (Pierce) according to the manufacturer’s instruction. Briefly, streptavidin coated magnetic beads were washed in wash buffers. 50 pmol RNA oligonucleotide probes labelled with 3′ biotin-tetra-ethyleneglycol spanning rs17651213 either H1-G (5’TGA GGG AGC TTT GCG TGT TTA TCC TCC TGT3’) or H2-A (5’TGA GGG AGC TTT GCA TGT TTA TCC TCC TGT3’), rs1800547 H1 (5’CCA CAG GUG AGG GUA AGC CCC AGA GAC CCC5’) or H2 (5’CCA CAG GUG AGG GUG AGC CCC AGA GAC CCC3’) (Integrated DNA Technology) were bound to the streptavidin coated magnetic beads and incubated with 40 μg SK-N-F1 nuclear enriched lysates. Unbound proteins were washed off. The magnetic beads together with the RNA bait and bound proteins were snap frozen and stored at -20 °C until further analysed.

### Mass spectrometry

Tryptic digests of RNA-bound proteins were analysed on an Ultimate 3000 RSLCnano HPLC (Dionex, Camberley, UK) system run in direct injection mode coupled to a QExactive Orbitrap mass spectrometer (Thermo Electron, Hemel Hempstead, UK). Samples were resolved on a 25 cm by 75 μm inner diameter picotip analytical column (New Objective, Woburn, MA, USA) which was packed in-house with ProntoSIL 120–3 C18 Ace-EPS phase, 1.9 μm bead (Bischoff Chromatography, Germany). The system was operated at a flow-rate of 250 nL min^−1^. A 120 min or 60 min gradient was used to separate the peptides. The mass spectrometer was operated in a “Top 20” data dependent acquisition mode. Precursor scans were performed in the orbitrap at a resolving power of 70,000, from which the 20 most intense precursor ions were selected by the quadrupole and fragmented by HCD at a normalised collision energy of 30%. The quadrupole isolation window was set at *1.6 m/z.* Charge state +1 ions and undetermined charge state ions were rejected from selection for fragmentation. Dynamic exclusion was enabled for 27 s. Data were converted from. RAW to. MGF using ProteoWizard [34].

### Mass spectrometry data analysis

Mass spectrometry. RAW data files for SNP rs17651213 were imported and processed with Progenesis QI for Proteomics software (Nonlinear Dynamics). The H1 probe sample data were selected as the alignment reference for all the replicates in the alignments of ion density maps. Automatic alignment was first performed followed by manual reviewing of the alignments. Peptide ions with a charge between 2 and 4 were included. Peptide abundance normalisation was performed automatically by the software. A between-subject design was set for analysed runs. Protein identification was performed using MS/MS ions search on the Computation Biology Research Group (Oxford University) Mascot Server (Matrix Science). Peptide ions were searched against the UPR_HomoSapiens database with fixed modifications set for carbamidomethyl at cysteine residues and variable modifications set for oxidation at methionine residues. Completed protein identifications were imported back into Progenesis QI for matching to their corresponding detected peptide ions. Proteins were quantified by Progenesis QI using a relative quantitation method using non-conflicting peptides.

Mass spectrometry. RAW data files for SNP rs1800547 were imported and processed with MaxQuant [35]. A minimum of two unique peptides were required to protein quantification, peptide abundances were normalised using the iBAQ algorithm. Peptide ions were searched against the UniProtHomo Sapiens database with fixed modifications set for carbamidomethyl at cysteine residues and protein identifications were obtained.

To identify splicing factors interacting with the RNA probes, proteins were matched to a list of 71 experimentally validated splicing factors obtained from the SpliceAid-F database (http://srv00.recas.ba.infn.it/SpliceAidF/) [36]. Candidate splice factors were manually curated by (Additional file 1: Table S4) excluding factors in the three experimental replicates where the H1/H2 protein abundance ratios were not consistently above or below 1 across replicates. At least two replicates were used in the final results. Splice factors with ratios of abundance between 1.2 and 0.8 were further excluded as candidates.

### Western blot

RNA pull-down fractions were denatured in Lamelli buffer (6X: 12% SDS, 30% β-mercaptoethanol, 60% glycerol, 0.012% bromophenol blue, 375 mM Tris pH 6.8) at 95 °C for 10 min. Following denaturation, proteins were separated by polyacrylamide gel at 200 V for ~45 min. Proteins were transferred using the Trans-Blot Turbo Transfer System (BioRad) onto polyvinylidene fluoride membrane contained in the Trans-Blot Turbo PVDF Transfer Packs (BioRad) according to the manufacturer’s protocol. Specific proteins were detected using anti-hnRNP F (IQ208 Immuquest) and anti-hnRNP Q (ab189405 Abcam) antibodies according to the manufacturer’s protocol.

Frontal cortex (BA 46) brain tissue was obtained from 5 H1/H2 PSP cases and 5 pathology-free controls from the brain banks of the Oxford Project to Investigate Memory and Ageing (OPTIMA) and the Thomas Willis Oxford Brain Collection. Brain tissue samples are collected with full consent of the patient and with the approval of the local Ethics Committee (COREC approval number 1656). Expression analysis has been approved by local Ethics Committee review (ref 06/Q1605/8). Total protein was extracted with an initial homogenization in 10 mls cold RIPA buffer per gram of tissue (RIPA 50 mM tris-HCl, pH 7.4, 150 mM NaCl, 1% (*v*/v) Triton X-100, 1% (*w*/*v*) sodium deoxycholate, 0.1% (w/v) SDS) with cOmplete mini protease inhibitors (Roche). The tissue was homegenized and sonicated before leaving on ice for 1 hour after which the soluble fraction was isolated by microcentrifugation (14,000 RPM, 10 mins, 4 °C) and Western Blotting ocucred as described above. Primary antibodies used for human brain samples: hnRNP Q (ab184946) 1: 10,000; hnRNP F (ab6095) 1:500. HRP-conjugated β-actin (ab49900) (1:20,000).

### GTEx data analysis

The Genotype-Tissue Expression (GTEx) Project was supported by the Common Fund (https://commonfund.nih.gov/GTEx) of the Office of the Director of the National Institutes of Health, and by NCI, NHGRI, NHLBI, NIDA, NIMH, and NINDS. The data used for the analyses described in this manuscript were obtained from the GTEx Portal on 08/17/2017: GTEx Analysis V6 (dbGaP Accession phs000424.v6.p1). Data analysis and visualisations were performed using R version 3.3.1 (https://www.r-project.org/). rs17650907 genotypes were used as a proxy for H1 and H2 haplotypes where H1 represent individuals with genotype AA and H2 with AG and GG. GTEx brain regions were grouped into cerebral cortex, cerebellum, amygdala, hippocampus, midbrain and spinal cord.

## Results

### Comparative sequence analysis of the genomic *MAPT* H1/H2 haplotype BAC and PAC vectors

To investigate haplotype-specific polymorphisms that may regulate the expression of alternatively spliced *MAPT* transcripts, we identified from the BACPAC resources (https://bacpacresources.org//) bacterial and P1-derived artificial chromosomes (BACs and PACs, respectively) which span the entire 143 kb *MAPT* gene. We genotyped the vectors at six haplotype-tagging polymorphisms used previously [37] and chose one H1 haplotype PAC (PAC61d06) [26] and one H2 BAC RP11-769P22 (accession BX544879) to take forward for our studies.

**Fig. 1:**
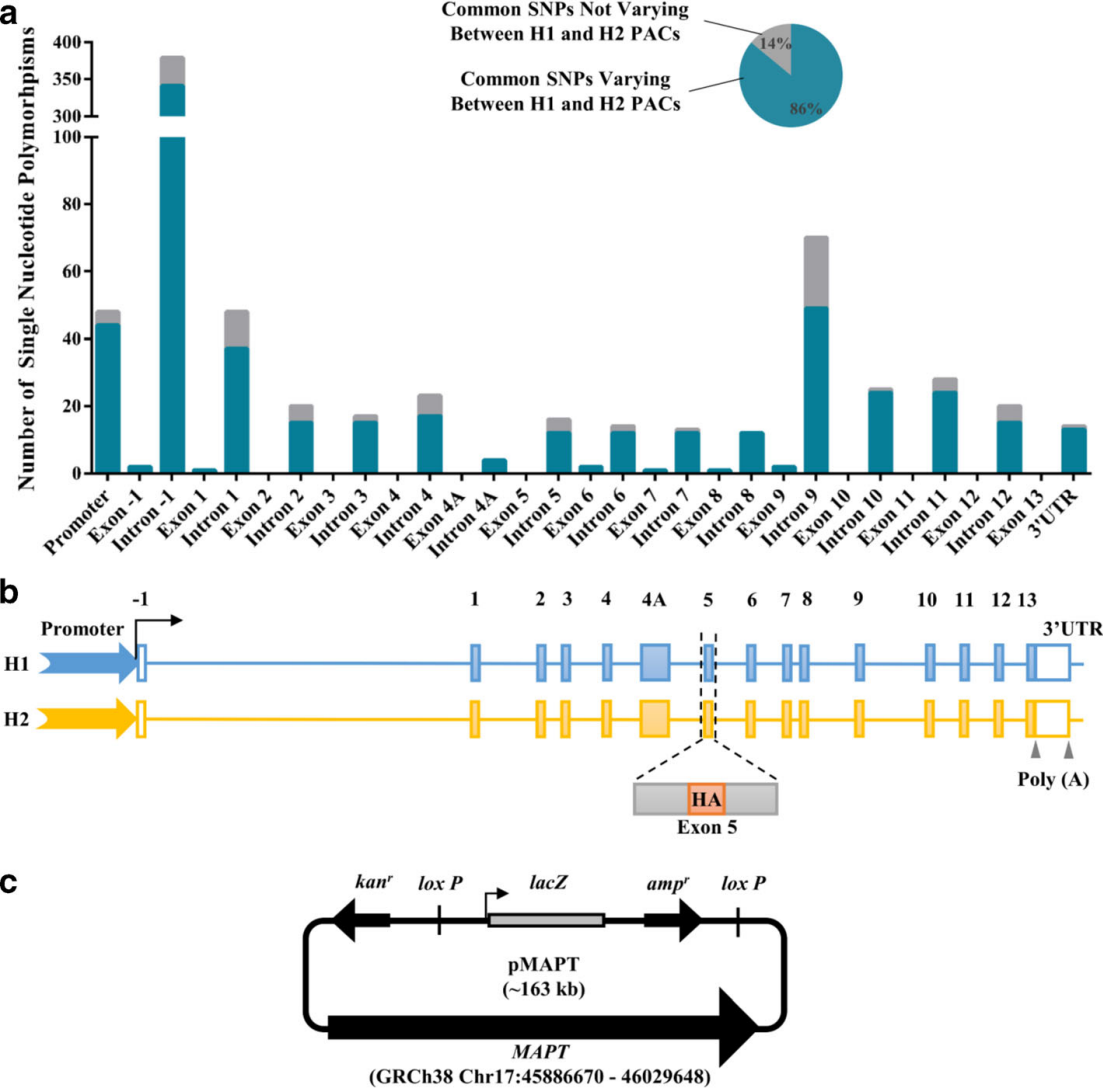
Whole genomic locus *MAPT* H1/H2 vectors capture over 86% sequence diversity. **a** Common SNPs as registered on the dbSNP b144 database identified between the H1 and H2 p*MAPT* vectors. **b** Schematic representation of the H1/H2 p*MAPT* genomic features. The H1 and H2 *MAPT* vector sequences start and end at the same point, and are not different between H1 and H2 except at the sites of haplotype-specific variation. The H1 and H2 sequences are comprised of promoter (coloured arrow), introns and exons (filled boxes and numbered), and 3′ untranslated region (3’UTR) and downstream sequences to include two polyadenylation sites (Poly (A)). (HA: haemagglutinin sequence, black arrow: transcription start, unfilled boxes: untranslated exons). **c** Schematic representation of the p*MAPT* vector. The *MAPT* sequence is ~143 kb. (lacZ: β-galactosidase gene, amp^r^ and kan^r^: ampicillin and kanamycin resistance)

To determine the coverage of common SNPs registered on the dbSNP b144 database by our H1 and H2 BAC/PAC vectors, we performed a detailed analysis on the 143 kb *MAPT* region contained in the vectors (Fig. 1a). Briefly, SNP data from the dbSNP database in the 143 kb *MAPT* region covered by our H1/H2 BAC/PAC vectors were used for comparisons between H1 and H2 sequences. We included only SNPs with a minor allele frequency of ≥5% as catalogued in the 1000 Genomes Project. A total of 659 common SNPs (Additional file 1: Table S5) were identified to be different between the H1 and H2 vectors, capturing over 86% of the common SNP diversity as registered on the dbSNP database in the corresponding *MAPT* region. Of the 655 SNPs, approximately 60% of the common SNPs are present in the upstream 7.7 kb promoter region and 67.7 kb 5′ untranslated intron −1 region (Fig. 1a). The cumulative 59.5 kb of intronic regions between coding exons harbour 38% of the identified SNPs. The remaining 2% SNPs locate in the 5.5 kb 3′ untranslated and downstream regions, and in coding exons (Fig. 1a).

Nine variants were identified in exons (Fig. 1a). Of these, SNP rs17650901 13 bp upstream of the translation start site in exon 1, and two synonymous SNPs rs1052553 and rs17652121 in exon 9, have been described previously [38]. Two SNPs rs11575895 and rs62056779 are present in the untranslated exon −1. Two additional synonymous SNPs occur in exons 7 (rs1052551) and 8 (rs62063845). Exon 6 contains two non-synonymous missense variants that result in serine to proline (rs10445337) and tyrosine to histidine (rs2258689) changes. We analysed the amino acid sequence of tau using SNAP (http://rostlab.org/services/snap/) [39], a program that makes predictions regarding the functionality of mutated proteins. The amino acid changes resulted from rs10445337 and rs2258689 in exon 6 were both predicted to have a neutral effect on protein function therefore unlikely to convey a haplotype-specific risk to neurodegeneration.

### Construction of comparative p*MAPT*-H1 and -H2 genomic expression vectors

To make direct comparisons of the functionality of sequences differing between H1 and H2 in a human neuroblastoma cell culture system, we generated two new vectors from a *MAPT*-BAC (generated from PAC61d06 in [26]) and RP11-769P22, named p*MAPT*-H1 and p*MAPT*-H2, respectively, by inserting the 143 kb H1 and H2 *MAPT* gene into identical PAC backbone plasmids. The p*MAPT*-H1 and –H2 vectors contain equivalent up and downstream regulatory regions, varying only at the sites of haplotype-specific variation. The start and end coordinates of the *MAPT* gene sequence correspond to chromosome 17:45,886,670–46,029,648 on the Genome Reference Consortium GRCh38 human genome assembly. The p*MAPT*-H1 and p*MAPT*-H2 vectors are comprised of 7.7 kb promoter sequences, the full complement of exons and introns, and 5.5 kb of downstream sequences sufficient to contain 2 polyadenylation sequences (Fig. 1b). The inclusion of all the regulatory elements of the *MAPT* gene is crucial for maintaining its physiological relevance for our studies on the effects of haplotype-specific polymorphisms on alternative splicing [26].

In order to specifically assay the expression of the transgenic p*MAPT* on the background of the endogenous *MAPT* in our human SK-N-F1 neuroblastoma cell culture model, we introduced a short haemagglutinin (HA) tag in-frame in exon 5 to act as a unique RT-PCR primer site. This site is found only in the transgene and therefore will report only transgene expression when used in QPCR assays (Additional file 1: Figure S1). Briefly, we used a selection/counter-selection method as previously described [26, 28, 40] to first introduce a streptomycin sensitive-chloramphenicol resistant (rpsl-chl) cassette into exon 5 followed by removal of the cassette and replacing it with the HA tag sequence (Fig. 1b). Correct constructs were identified by restriction enzyme digestion and pulsed-field gel electrophoresis, followed by verification using sequencing. The p*MAPT* vectors were then retrofitted with pH-FRT-Hy plasmid [41] by Cre-mediated loxP recombination to incorporate a β-galactosidase (*lacZ*) reporter gene (Fig. 1c) [41].

### Polymorphisms in exon 3 flanking regions represent haplotype-specific splice factor binding sites

**Fig. 2:**
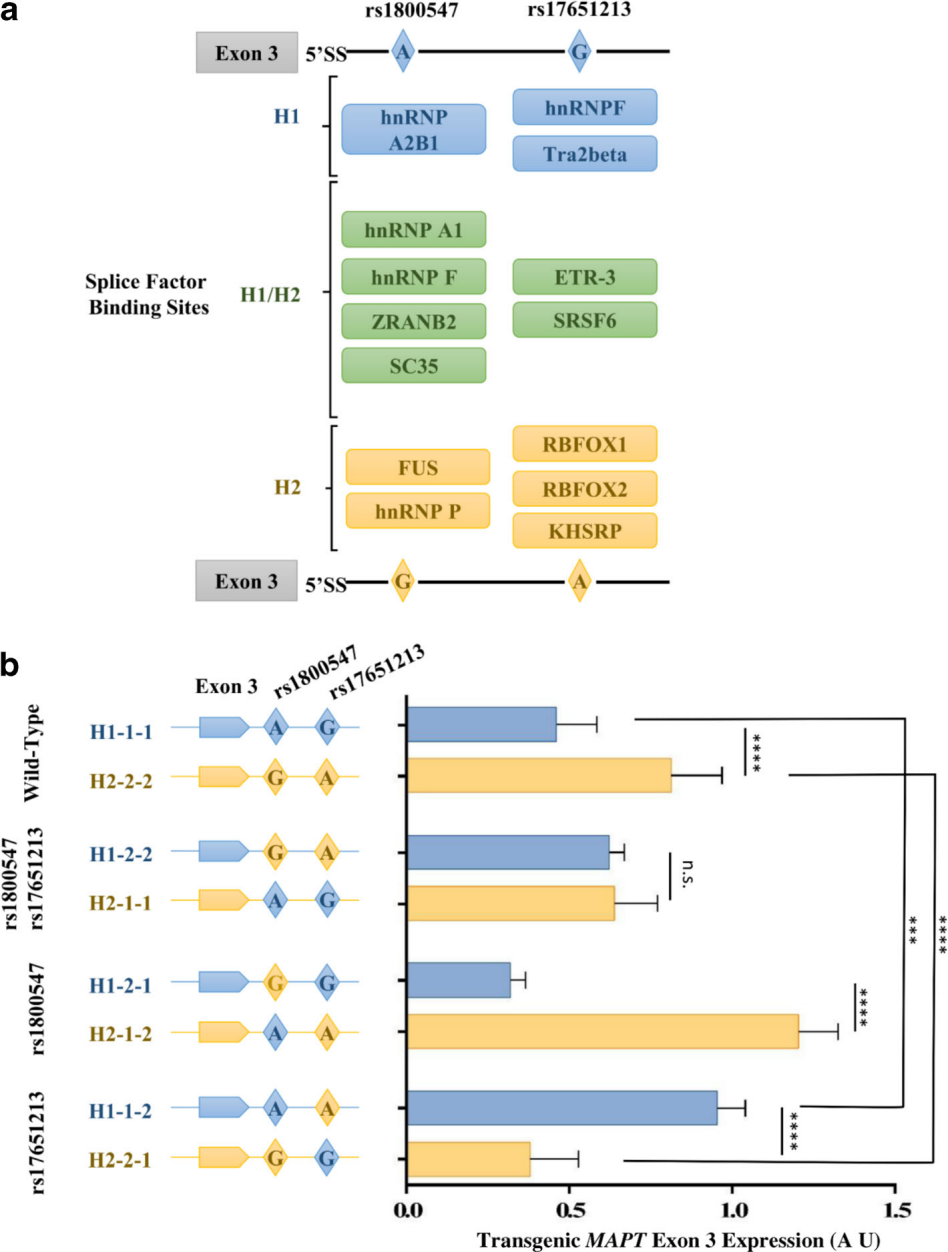
rs1800547 and rs17651213 regulates the haplotype-specific inclusion of *MAPT* exon 3. **a** Splice factor binding site prediction using three different online tools: SFmap, SpliceAid2 and ESE. H1/H2 alleles from SNP rs1800547 and rs17651213 both feature haplotype-specific splice factor binding sites and share common binding sites. (5′SS: 5′ splice site, 3′SS: 3′ splice site). **b** Left panel: a schematic representation illustrating the combinations of SNPs rs1800547 and rs17651213 exchanged between the H1 and H2 p*MAPT* vectors. Right panel: the corresponding p*MAPT* exon 3 expression in SK-N-F1 neuroblastoma cells as measured by qPCR. Exon 3 expression from the H2WT allele (0.81 ± 0.16, *n* = 8) was 1.76 fold higher when compared to the H1WT allele (0.46 ± 0.12, *n* = 8). No difference in exon 3 expression was observed between the H1*–2-2* (0.62 ± 0.05, *n* = 4) and H2*–1-1* (0.64 ± 0.13, *n* = 4) vectors. Exon 3 expression was 3.78 fold higher from the H2*–1-2* (1.20 ± 0.12, *n* = 4) vector when compared to the H1*–2-1* (0.32 ± 0.05, *n* = 4) vector. Exon 3 expression was 2.52 fold higher from the H1*–1-2* (0.95 ± 0.09, *n* = 4) vector when compared to the H2*–2-1* (0.38 ± 0.15, *n* = 4) vector. Statistical significance was determined using one-way ANOVA followed by Bonferroni correction (*** *p* < 0.001, **** *p* < 0.0001). Error bars represent standard deviation

To identify candidate polymorphisms that may be responsible for the increased inclusion of exon 3 in H2 transcripts, the H1 and H2 sequences upstream and downstream of exon 3 were aligned using the programme ApE (http://biologylabs.utah.edu/jorgensen/wayned/ape/). In total, 15 haplotype variant SNPs were identified in intron 2 (between exons 2 and 3), and 15 SNPs in intron 3 (between exons 3 and 4). Of these, none was identified within the splice sites, predicted branch points or polypyrimidine tract (http://www.umd.be/HSF3/index.html) [42, 43]. However, it has been shown that polymorphisms closest to the exon-intron boundaries are those most often associated with an alternative splicing phenotype [44]. We therefore analysed 2 variants (rs1800547 and rs17651213) that fall within 100 bp downstream of exon 3 using SFmap, SpliceAid2 and ESEfinder to predict the binding sites of common splice factors [45–47]. We identified putative splice factor binding sites at the rs1800547 and rs17651213 SNP sequences that can be separated into those predicted for both H1 and H2 haplotypes and those unique to either H1 or H2 (Fig. 2a). The results suggest that altered splice factor binding sequence by haplotype-specific SNPs could be a potential mechanism of regulating allele-specific inclusion of *MAPT* exon 3. The two candidate SNPs, rs1800547 and rs17651213, were found to be in strong LD with the H1/H2 haplotype structure (Additional file 1: Figure S2). In addition, they are in close proximity to the exon 3–5′ splice site and the putative haplotype-specific binding of splice factors. We therefore selected these variants as potential functional variants for further study in our BAC PAC models.

### Generation of p*MAPT*-H1 and -H2 haplotype-hybrid genomic expression vectors

Genomic locus expression vectors provide an excellent tool for assaying the effects of intronic sequence variants on alternative splicing. To assess the functionality of the two selected candidate SNPs rs1800547 and rs17651213, we used the selection/counter-selection method described above to generate haplotype-hybrid vectors in which the allele from the H1 haplotype has been engineered onto the H2 background and vice versa. In total we generated 4 pairs of p*MAPT* haplotype-hybrid vector sets, including the wild-type vectors. The vectors were generated with the following combinations of SNPs swapped between the haplotypes: i) wild-type vectors H1–*1-1* and H2–*2-2*, ii) both 1,800,547 and rs17651213 swapped (H1–*2-2* and H2–*1-1*), iii) rs1800547 swapped (H1–*2-1* and H2–*1-2*) and iv) rs17651213 swapped (H1–*1-2* and H2–*2-1*) (Fig. 2b). All the vectors carry a *lacZ* gene in the pH-FRT-Hy plasmid backbone [41]. The expression of the reporter gene was used as a means to assess the efficiency of vector delivery into cultured cell models (Additional file 1: Figure S3).

### rs17651213 regulates the haplotype-specific expression of *MAPT* exon 3–containing transcripts in SK-N-F1 neuroblastoma cells

We first determined in our SK-N-F1 neuroblastoma culture model whether the H1 and H2 wild-type p*MAPT* vectors (Fig. 2b) recapitulate human physiological expression. We examined GTEx data to look at transcripts with and without exon 3(Additional file 1: Figure S4). We see individuals with an H2 allele have significantly greater exon 3(+) transcripts in the cerebellum, cerebral cortex, hippocampus and midbrain than individuals without an H2 allele. This effect which does not occur with exon 3(−) transcripts. This data is in agreement with previous results showing the H2 allele expresses two-fold higher exon 3-containing transcripts when compared to H1 [19, 48] We quantified the expression levels of exon 3-containing transcripts in comparison to total *MAPT* expression, as determined by qPCR, from our H1 and H2 wild-type vectors in SK-N-F1 neuroblastoma cells (Fig. 2b). We observed a 1.76 fold higher exon 3 inclusion from the H2 wild-type vector when compared to the H1 (H2–*2-2*: 0.81 ± 0.16 and H1–*1-1*: 0.46 ± 0.12; *p* < 0.0001) (Fig. 2b), indicating that exon 3 inclusion from our genomic DNA haplotype expression vectors is representative of the endogenous *MAPT* exon 3 expression previously reported [19, 48].

We next aimed to determine the effects of the candidate haplotype-specific SNPs on exon 3 expression in the SK-N-F1 cell culture model by using our hybrid vectors (Fig. 2b). We expressed the H1 and H2 vector pair (H1–*2-2* and H2–*1-1*) with both rs1800547 and rs17651213 exchanged between the haplotype backgrounds and found exon 3 expressed to the same level between the pair of vectors (H1–*2-2*: 0.62 ± 0.05 and H2–*1-1*: 0.64 ± 0.13), suggesting either one or both SNPs contribute to the haplotype-specific expression pattern of exon 3 (Fig. 2b).

To study the effects of the SNPs rs1800547 and rs17651213 on exon 3 expression individually, we expressed the vector pair with only rs1800547 exchanged between the H1/H2 backgrounds. SNP rs1800547 is located 9 bp downstream of the 5′ splice site of exon 3 and is expected to have a strong effect on the inclusion or exclusion of exon 3. Indeed, exchanging rs1800547 led to a 3.78 fold greater expression of exon 3 from the H2 allele when compared to H1 (H2–*1-2*: 1.2 ± 0.12 and H1–*2-1*: 0.32 ± 0.05; p < 0.0001), further increasing the inclusion of exon 3 from the H2 background compared to the wild-type expression (Fig. 2b). Expressing the vector pair with only rs17651213 exchanged between the H1/H2 backgrounds resulted in a 2.52 fold higher exon 3 expression from the H1 allele compared to the H2 (H1–*1-2*: 0.95 ± 0.09 and H2–*2-1*: 0.38 ± 0.15; p < 0.0001) (Fig. 2b).

Together, the expression of exon 3-containing transcripts from different genomic-hybrid p*MAPT* vectors in the SK-N-F1 neuroblastoma cell culture model indicates that rs1800547 and rs17651213 are both functional SNPs that contribute to allelic differences in exon 3 expression. The reversal of the wild-type exon 3 expression profiles by vectors H1–*1-2* and H2–*2-1* (Fig. 2b) strongly suggests that rs17651213 confers the haplotype-specificity for expression of *MAPT* exon 3.

### Allele-specific RNA-protein complex formation by H1/H2 rs1800547 and rs17651213 sequences​

**Fig. 3:**
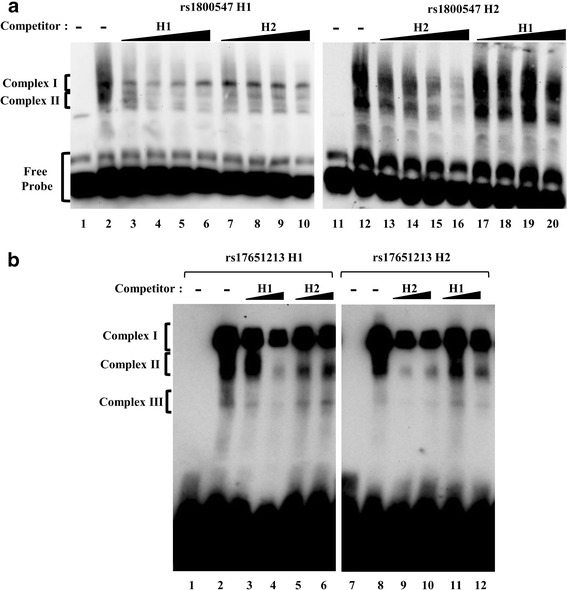
Allele-specific RNA-protein complex formation by H1/H2 rs1800547 and rs17651213 sequences (**a**) RNA-protein complex formation by rs1800547 H1 (lane 2) and H2 (lane 12) biotin-labelled RNA probes with SK-N-F1 nuclear extract by RNA-EMSAs. The displacement of RNA-protein complex formation by unlabelled rs1800547 H1 (lanes 3–6, 17–20) or H2 (lanes 7–10, 13–16) competitor RNA probes of 10X, 20X, 40X and 100X in molar excess. The absence of SK-N-F1 nuclear extract as a negative control for RNA-protein complex formation (lanes 1 & 11). **b** RNA-protein complex formation by rs17651213 H1 (lane 2) and H2 (lane 8) biotin-labelled RNA probes with SK-N-F1 nuclear extract by RNA-EMSAs. The displacement of RNA-protein complex formation by unlabelled rs17651213 H1 (lanes 3, 4, 11, 12) or H2 (lanes 5, 6, 9, 10) competitor RNA probes of 500X and 1000X in molar excess. The absence of SK-N-F1 nuclear extract as a negative control for RNA-protein complex formation (lanes 1 & 7)

We next wished to investigate the *trans*-acting splicing factors that interact with SNPs rs1800547 and rs17651213. To understand the mechanisms underlying the allele-specific regulation of *MAPT* exon 3 inclusion identified from the genomic *MAPT* vector study above, we performed RNA-EMSAs (RNA electrophoretic mobility shift assays) to study the impact of the SNP sequences on RNA-protein complex formation. RNA-protein complex formation by biotin-labelled RNA probes, containing either H1 or H2 rs1800547 or rs17651213 sequences, with proteins in the form of nuclear extract from SK-N-F1 cells, were visualised as “gel shift” in RNA-EMSAs (Fig. 3a lanes 2 & 12, 3B lanes 2 & 8). The H1 and H2 RNA-protein complexes formed by SNPs rs1800547 (Fig. 3a lanes 2 & 12) exhibit different intensities in the complex profiles, indicating that the H1/H2 alleles contribute to altering the composition of RNA-protein complexes formed by the SNP in vitro.

We further compared the binding specificities of the rs1800547 and rs17651213 RNA-protein complexes by the H1 and H2 alleles (Fig. 3a lanes 2–6 & 8–12, 3B lanes 2–6 & 8–12) in our competition RNA-EMSA experiments using increasing amounts of either H1 or H2 unlabelled RNA probes to compete with the labelled probes for protein binding. The competition strength of the unlabelled RNA probes were determined by assessing the intensities of RNA-protein complexes formed where the more the RNA-protein complex is displaced, the higher the competition strength. We observed allele-specific RNA-protein interaction by the H2 allele of rs1800547, and both H1 and H2 rs17651213 alleles, where competitor sequences of a different allele were less capable of outcompeting RNA-protein complex formation with that of the same allele (Fig. 3a lanes 12–20, 3B lanes 2–6 & 8–12).

The observed H1/H2-specific RNA-protein interactions by rs1800547 and rs17651213 demonstrate that changes to a cis-element by a single base change leading to altered recognition by trans-acting splice factors could form the basis of regulating *MAPT* exon 3 inclusion by common intronic variants.

### Identification of splicing factors interacting with rs17651213 and rs1800547

Under the binding conditions required for EMSA, we showed that there are differences in protein species binding to our H1 and H2 RNA probes for rs17651213 and rs1800547. However, this does not provide information about which proteins are specifically binding to our region of interest. We therefore undertook to identify the splice factors that bind these regions using RNA-protein pull down followed by mass spectrometry. Briefly, RNA oligonucleotide probes spanning the rs17651213 or the rs1800547 alleles were incubated with SK-N-F1 nuclear enriched lysates and RNA binding proteins were then identified using label-free mass spectrometry. We confirmed the presence of the proteins in the RNA pull-down fractions using Western Blot (Additional file 1: Figure S5). Proteins identified either with low abundance or with a lack of reproducibility between experimental replicates were excluded from the results. For the identification of splice factors, the list of candidate proteins were matched against a list of 71 known human splice factors obtained from the SpliceAidF database [36]. The relative abundance of splicing factors binding to the H1 and H2 alleles were compared (Tables 1 and 2).

**Table 1:** Identifying candidate splice factors interacting with H1 and H2 rs17651213 alleles

Gene Symbol	Description	Ratio H1:H2	±S.D.
SFPQ	Splicing Factor Proline And Glutamine Rich	2.4	0.5
TARDBP	TAR DNA Binding Protein	2.2	0.1
HNRNPM	Heterogeneous Nuclear Ribonucleoprotein M	2.0	0.3
ELAVL1	ELAV Like RNA Binding Protein 1	2.0	0.1
HNRNPH3	Heterogeneous Nuclear Ribonucleoprotein H3	1.9	0.3
HNRNPA0	Heterogeneous Nuclear Ribonucleoprotein A0	1.7	0.2
PTBP1	Polypyrimidine Tract Binding Protein 1	1.7	0.0
hnRNPQ	Heterogeneous Nuclear Ribonucleoprotein Q	1.6	0.5
HNRNPA2B1	Heterogeneous Nuclear Ribonucleoprotein A2/B1	1.6	0.3
PTBP2	Polypyrimidine Tract Binding Protein 2	1.3	0.2
KHDRBS3	KH RNA Binding Domain Containing, Signal Transduction Associated 3	1.3	0.1
HNRNPF^a^	Heterogeneous Nuclear Ribonucleoprotein F	1.2	0.1

H1 and H2 rs17651213 RNA-pull down of SK-N-F1 nuclear proteins followed by mass spectrometry identification. The values of the mean abundance ratio of proteins interacting with H1: H2 probes were reported with the standard deviation (S.D.). ^a^Proteins predicted to bind the H1 and/or H2 probe sequences by SFmap, ESE and SpliceAid2. Splice factors exhibit preferential bindings of 20% or above to either H1 or H2 alleles

**Table 2:** Identifying candidate splice factors interacting with H1 and H2 rs1800547 alleles

Gene Symbol	Description	Ratio H1:H2	±S.D.
KHDRBS3	KH RNA Binding Domain Containing, Signal Transduction Associated 3	3.1	0.6
ELAVL1	ELAV Like RNA Binding Protein 1	2.6	1.4
PCBP1	Poly(RC) Binding Protein 1	2.2	0.1
RBMX	RNA Binding Motif Protein, X-Linked	2.1	0.7
HNRNPK	Heterogeneous Nuclear Ribonucleoprotein K	1.5	0.2
HNRNPU	Heterogeneous Nuclear Ribonucleoprotein U	1.5	0.2
HNRNPA2B1^a^	Heterogeneous Nuclear Ribonucleoprotein A2/B1	1.5	0.4
PCBP2	Poly(RC) Binding Protein 2	1.4	0.2
KHDRBS1	KH RNA Binding Domain Containing, Signal Transduction Associated 1	0.8	0.1
SFPQ	Splicing Factor Proline And Glutamine Rich	0.8	0.3
HNRNPM	Heterogeneous Nuclear Ribonucleoprotein M	0.8	0.2
SRSF3	Serine And Arginine Rich Splicing Factor 3	0.8	0.3
SF3B1	Splicing Factor 3b Subunit 1	0.7	0.3
hnRNPQ	Heterogeneous Nuclear Ribonucleoprotein Q	0.3	0.2
PTBP1	Polypyrimidine Tract Binding Protein 1	0.2	0.2

H1 and H2 rs17651213 RNA-pull down of SK-N-F1 nuclear proteins followed by mass spectrometry identification. The values of the mean abundance ratio of proteins interacting with H1: H2 probes were reported with the standard deviation (S.D.). ^a^Proteins predicted to bind the H1 and/or H2 probe sequences by SFmap, ESE and SpliceAid2. Splice factors exhibit preferential bindings of 20% or above to either H1 or H2 alleles

At rs17651213,alleles of which showed greatest ability confer an exon 3 splicing expression similar to their haplotype of origin, we identified 12 splice factors which reproducibly demonstrated differential (>20%) binding to the H1 or H2 alleles (Table 1). The number of peptides used for abundance quantification of the candidate splice factors was highly consistent between experimental replicates (Additional file 1: Table S6 and S7). Notably, hnRNPF, which was predicted to interact with rs17651213 H1 allele (Fig. 2), is among the factors identified with a H1:H2 binding ratio of >1.2.

At rs1800547, there were 15 factors which showed a 20% or greater difference in binding the two alleles. Unlike rs17651213 factors which displayed H1:H2 binding ratios greater than 1, rs1800547 had approximately equal numbers of splice factors with ratios greater and less than 1 (Table 2). hnRNP A2B1 was predicted to bind the rs1800547 H1 allele and has a H1:H2 ratio of 1.5. Identification of proteins that differentially bind the alleles of rs17651213 and rs1800547 demonstrates that the change of one nucleotide can change the splice factors recruited to intronic regions.

To confirm if a functional effect on splicing is caused by this differential binding of splice factors, we selected nine splice factors that displayed differential binding at rs17651213 to determine their effect on allele-specific *MAPT* exon 3 expression due to the strength of the variation effect observed using our genomic locus expression vectors (Fig. 2b).

### Development of a quantitative *MAPT* allele-specific expression assay

In order to quantify the endogenous H1 to H2 *MAPT* exon 3 transcript ratios in the H1/H2 heterozygous SK-N-F1 cells, we developed a Taqman based allele-specific qPCR assay. We designed H1 and H2 specific Taqman probes such that they bind to either the H1 or the H2 allele of a common H1/H2 polymorphism rs17650901 in exon 1 (Additional file 1: Figure S6A). The quantitation of H1 and H2 transcripts were measured by FAM and VIC fluorescence signals, respectively, using qPCR (Additional file 1: Figure S6B). By mixing the H1 and H2 p*MAPT* vectors in the ratios 8:1, 4:1, 2:1, 1:1, 1:2, 1:4 and 1:8 (Additional file 1: Figure S4B), we generated a standard curve where the delta Ct values between FAM (H1) and VIC (H2) signals were plotted against the log_2_ ratios of H1:H2 transcripts (Additional file 1: Figure S4C). The standard curve has an r^2^ value of 0.9979, where log_2_ (H1/H2) = (−1.194 × delta Ct) + 0.5615, indicating a linear relationship between the delta Ct values and the log_2_ ratios of H1:H2 transcripts. Since a linear relationship is observed here and the slope is constant regardless of the y-intercept, we used the equation of log_2_ (H1/H2) = −1.194 × delta Ct for the log_2_ ratios of H1:H2 transcripts from measured delta Ct values between the H1 (FAM) and H2 (VIC) probes. siRNA knockdown of hnRNP Q and hnRNP F increased H1:H2 *MAPT* Exon 3 transcript ratios.

**Fig. 4:**
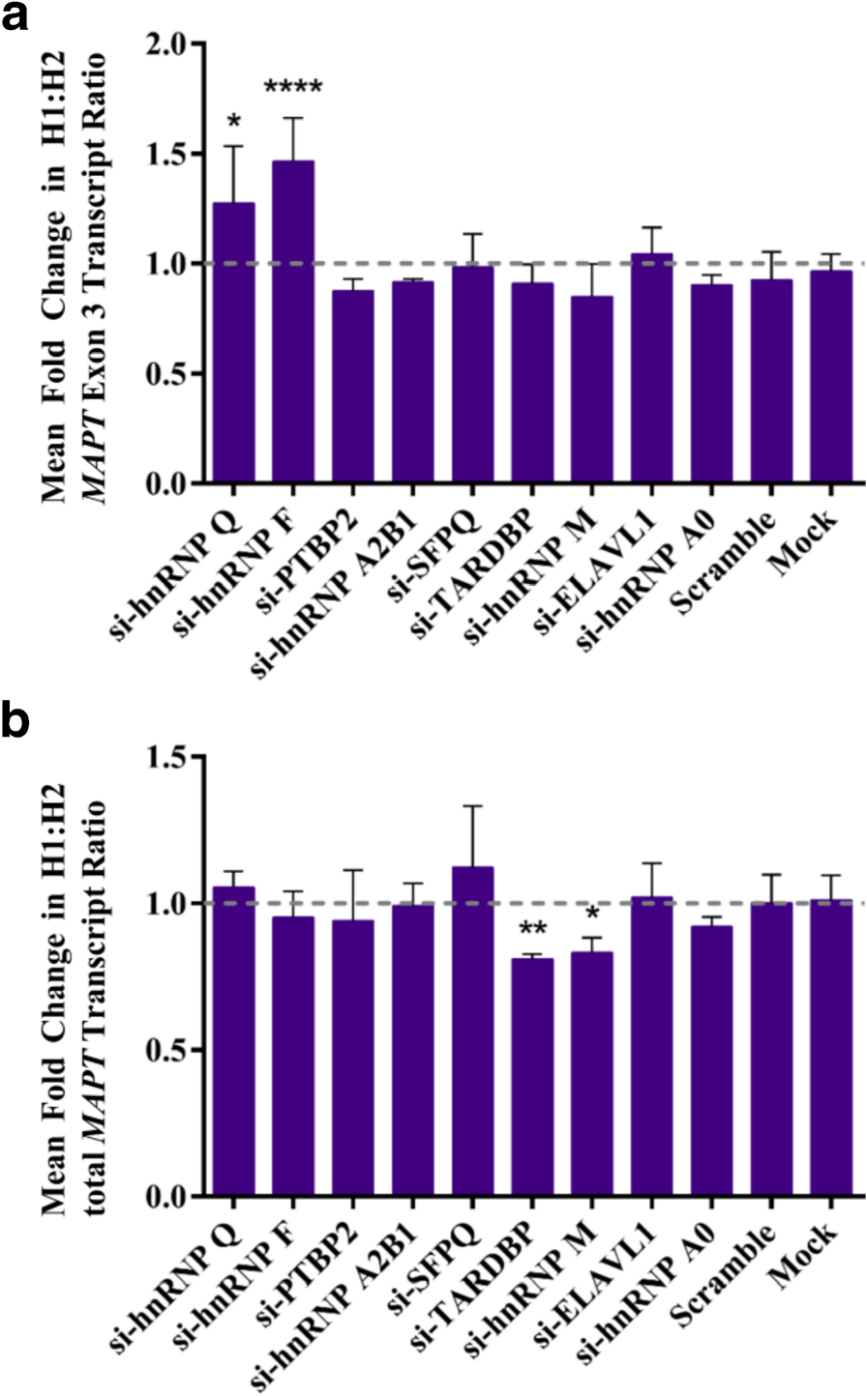
hnRNP Q and hnRNP F regulate the haplotype-specific inclusion of *MAPT* exon 3 (**a**) Mean fold change in the normalised H1:H2 *MAPT* exon 3 transcript ratios in SK-N-F1 cells transfected with siRNA against different splice factors (*n* = 3–6), a negative siRNA control and a mock transfection (*n* = 8). hnRNP F (1.47 ± 0.19, *p* < 0.0001), hnRNP Q (1.28 ± 0.26, *p* < 0.05). Statistical significance was determined by comparing each column to the mock control (0.96 ± 0.08) using one way ANOVA with Bonferroni correction. Error bars represent standard deviation. **b** Mean fold change in the normalised H1:H2 total *MAPT* transcript ratios in SK-N-F1 cells transfected with siRNA against different splice factors

To determine the allele-specific effects of nine candidate splice factors, on exon 3 expression, we knocked down the expression of the splice factors using commercially available siRNAs in SK-N-F1 cells (Additional file 1: Figsure S7 & S8). We then employed the Taqman based allele-specific expression assay above to measure the ratios of H1:H2 *MAPT* exon 3-containing transcripts in our knockdown experiments. Here, we observed a significant increase in the H1:H2 exon 3 transcript ratios when hnRNP Q (H1:H2 1.28 ± 0.26, *p* < 0.05) and hnRNP F (H1:H2 1.47 ± 0.19, *p* < 0.0001) were knocked down compared to the mock transfection control (Fig. 4a). The H1:H2 total *MAPT* transcript ratios were not found to be significantly altered when hnRNP Q and hnRNP F were silenced (Fig. 4b), indicating that the increase in H1:H2 exon 3 transcript ratio in hnRNP Q and hnRNP F silencing is exon 3 specific and does not result from a change in total *MAPT* H1:H2 transcript ratio (Additional file 1: Figure S9). We confirmed the increase in H1:H2 exon 3 transcript ratios when hnRNP F and hnRNP Q in another heterozygous neuroblastoma cell line, SK-N-MC (F: H1:H2 1.74 ± 0.46, p < 0.05; Q: H1:H2 1.77 ± 0.13 p < 0.05) providing evidence that the haplotype-specific effects we see are not specific to SK-N-F1 (Additional file 1: Figure S10). hnRNP F and hnRNP Q are detected in human brain at the protein (Additional file 1: Figure S11) and transcript level (Additional file 1: Figure S12). The transcript levels of these splice factors are poorly correlated with exon 3 inclusion (Additional file 1: Figure S13 & S14), which suggests the impact these factors may have is through interactions with the specific haplotype sequences. Here, we showed that a reduction in hnRNP Q and hnRNP F levels lead to an increase in *MAPT* H1 exon 3-containing transcripts relative to the H2, suggesting that the two splice factors are normally involved in relative enhancement of H2 *MAPT* exon 3 inclusion.

## Discussion

In this study, we have combined whole genomic locus expression vectors with RNA-Protein identification and validation to identify functional variants that alter the expression of *MAPT* exon 3 through interaction with protein splice factors. These combined techniques have enabled us to assay the effect of risk associated variants within a large region of LD. By using haplotype-hybrid *MAPT* genomic locus vectors in a cell culture model, we have identified a functional variant rs17651213 that imparts a two-fold increase in H2-*MAPT* exon 3 expression compared to H1, a haplotype-specific expression pattern which has previously observed in both cell culture and in post-mortem brain tissue [19, 48]. Additionally, we identified rs1800547 which, also alters the regulation of the H1/H2 haplotype-specific alternative splicing of exon 3, although does not confer the splice phenotype of its haplotype background. Furthermore, we use mass spectrometry to identify splice factors that differentially bind to these alleles and confirmed that hnRNP Q and hnRNP F, two factors that displayed differential binding to rs17651213 alleles, alter the expression of *MAPT* exon 3 from the two haplotypes. Importantly, the haplotype-specific exon 3 inclusion by rs17651213 H1/H2 variants is highly dependent on the presence of either the H1 or H2 variant of its upstream functional SNP rs1800547, demonstrating the complex interactions between the functional SNP and its surrounding haplotype sequence context.

Experimental evidence supports roles for numerous factors in splicing including RNA-protein interactions, epigenetic regulation, co-transcriptional splicing, RNA secondary structures and RNA quality control systems (reviewed in [49]). Furthermore, the recognition motifs are short and motifs are short and degenerate and can be recognized by multiple different proteins, which in turn form complexes that have the ability to alter binding affinities and specificities of their peers [50]. The combination of these factors to regulate splicing contributes to the complexity of splicing regulation. The data we present here identifies just some of the cis-elements and trans-acting factors that contribute to the splice phenotype of *MAPT* exon 3. The context specific nature of the cis-element roles in splicing is demonstrated through the interactions of the alleles rs1800547 and rs17651213, which alone can enhance the haplotype-splice phenotype seen at exon 3, but when combined serve to act against the enhancing or silencing activity of the other SNP. Here we have limited our investigations to identifying polymorphisms and trans-acting factors that are contributing to the allele-specific expression of exon 3, focusing predominately on rs17651213 as the example of a cis-acting element that could convey the haplotype-specific expression profile of exon 3 when swapped on genetic backgrounds however there remains great scope for additional investigations.

Multiple GWAS and subsequent meta-analyses consistently report the H1 and H2 *MAPT* haplotypes to be over and under-represented, respectively, in PD, PSP and CBD [13–15], demonstrating the genetic risk and protection contributed by the H1 and H2 polymorphisms. Dissecting the mechanistic effects of H1/H2 polymorphisms that lead to splicing changes therefore requires methods that can encompass the large genomic linkage disequilibrium structures.

We present here a novel application for whole genomic locus vectors to study the functional effects of genetic variations on alternative splicing. Previously, mini-gene splicing constructs have been used to identify functional sequences and study mutations in alternative splicing [51]. However, in order to understand the functional significance of SNPs, there is a great benefit to using whole genomic locus vectors where the full complement of haplotype-specific polymorphisms in the non-coding regions, including all introns, upstream and downstream sequences of a gene, can be captured and manipulated. Our p*MAPT*-H1 and –H2 wildtype genomic DNA vectors carrying the 143 kb *MAPT* locus recapitulate the expression of endogenous exon 3, which is expressed at a two-fold higher level from the H2 allele compared to the H1, providing the correct physiological context of genetic variations from which they can be modified and studied. We have achieved single base pair accuracy in manipulating genomic DNA vectors, thereby allowing us to identify the precise haplotype-specific functions of rs1800547 and rs17651213 on exon 3 inclusion. Recent genome wide analyses of genetic variations showed SNPs are often associated with differences observed in gene expression and splicing [52, 53]. More importantly, SNPs in strong LD with lead risk SNPs identified in GWAS are often enriched in regulatory elements [7, 11], illustrating the importance of understanding the functions of non-coding sequence variations. Here, genomic DNA vectors that are able to capture this sequence diversity provide a novel and powerful tool to study differential regulation of gene expression and alternative splicing by SNPs, both in normal physiology and disease associations.

In silico analyses provide informative data that suggest potential mechanisms of differential exon 3 inclusion by rs1800547 and rs17651213 SNP sequences as the H1 and H2 alleles were predicted to bind different splice factors. We postulate that H1/H2 SNPs may regulate exon 3 inclusion by generating new splice factor binding sites and/or by altering the sequence strength for splice factor binding, two mechanisms that are not mutually exclusive. Previous studies have shown poor correlation between sequence motif predictions and RNA or DNA-protein interaction events [9, 54]. In vitro validations of in silico RNA-protein interaction predictions are therefore important in interrogating the mechanisms of splicing regulation. Our RNA-EMSA and RNA-protein pull-down experiments showed variant sequences confer allele-specific RNA-protein interactions and differences in sequence strengths for splice factor bindings, further supporting the notion that the H1/H2 SNPs modulate haplotype-specific exon 3 splicing by altering RNA-protein interactions. DNA/RNA-affinity approaches provide an unbiased means of studying nucleic acid and protein interactions [55], while label-free quantification of peptides provides a flexible method for comparing protein abundance in different samples [56]. Here, we discovered a trans-acting splicing regulator hnRNP Q interacting with SNP rs17651213 by RNA-protein pull-down experiments which was not previously predicted by computational sequence analysis based on consensus motifs. DNA/RNA-affinity approaches thus provide an informative method to screen for and to identify new RNA-protein interacting partners for further functional studies. hnRNP F was identified to interact with SNP rs17651213 in our RNA-protein pull-down. Our data highlight the significance of complementing computation predictions with biological data for identifying true RNA/DNA-protein interaction events.

We developed an allele-specific expression assay which allowed us to study changes in the H1:H2 transcript ratio following knockdown of splicing factors. We found the silencing of both hnRNP Q and hnRNP F led to an increase in the H1:H2 exon 3 *MAPT* transcript ratio, indicating that they promote the exclusion and/or inclusion of exon 3 from the H1 and H2 alleles, respectively, under normal conditions. Previous studies have demonstrated that the regulation of inclusion of exons by splicing factors is highly context specific. The same cis-regulatory sequence element could have both enhancer and silencer effects depending on the surrounding sequences. For example, deletion experiments of hnRNP F binding motifs (G-run elements) in the fibrinogen gamma-chain gene pseudoexon showed that the deletion of a silencer G-run element could have enhancing effects on the pseudoexon if neighbouring G-run elements are not present [54]. Likewise, the same splice factor could promote both exon inclusion and skipping, depending on the sequence context. For example, recent genome wide analyses on alternative splicing events showed that the depletion of hnRNP F proteins led to both activation and repression of alternative exons, strongly indicating that hnRNP F normally regulates both the enhancing and silencing of alternative exons [57, 58]. The interaction between rs1800547 and rs17651213 and their individual effect on exon 3 inclusion are likely to be complex and highly reliant on surrounding sequencesince exon 3 has an intrinsically suboptimal branch point at the 3′ splice site [59]. Nevertheless, our data from our p*MAPT* haplotype-hybrid vector study highlight the haplotype-specific differences between H1 and H2 SNP alleles and their combinatorial effects on regulation exon 3 inclusion.

The strong association of rs1800547 and rs17651213 with the PD GWAS risk SNP rs17649553 (Additional file 1: Figure S1) and the functional effects of on the two haplotype-specific SNPs on exon 3 inclusion could be contributing to the risk or protection conferred by the H1 and H2 haplotypes respectively. Exon 3 encodes the N-terminal acidic projection domain that mediates the interaction of tau protein with various cellular components such as the plasma membrane, dynactin, actin cytoskeleton, phospholipase C-γ and tyrosine kinase fyn signalling pathways and axonal transport processes [60–69]. many of which are implicated in PD pathogenesis [70–72]. The 2N tau protein isoform interacts with preferentially with proteins which map to neurodegenerative disease pathways such as AD, PD and Huntingdon’s disease [24]. In some neuropathology studies, 2N tau protein does not stain tau inclusions in CBD post-mortem brain tissue [73], and blots of sarkosyl-insoluble tau from both PSP and CBD lack 2N Tau isoforms [74], though we note studies using different conditions have been also detect 2N tau in CBD and PSP. There is evidence that 2N isoforms depress tau aggregation [25] which may indicate a route by which 2N tau offers some protection from disease. In this study we have investigated the genetic mechanisms which regulate the inclusion of exon 3 under haplotype-specific control. Understanding how different exon 3 tau isoform levels mediate processes implicated in neurodegeneration will provide further insight into the mechanisms of H1/H2 polymorphisms confer risk/protection in neurodegeneration.

## Conclusions

This work demonstrates an integrated approach to characterising the functionality of risk variants in large regions of linkage disequilibrium. Firstly, this approach uses whole genomic locus expression vectors to identify candidate functional variants, and subsequently interrogates these candidates with biochemical methodologies to identify splice factors with differential allelic binding. Applying these methodologies, we have identified common splice factors, hnRNP F and hnRNP Q, which regulate the haplotype-specific splicing of *MAPT* exon 3 by differential allelic-binding to intronic variants rs1800547 and rs17651213. *MAPT* exon 3 inclusion in transcripts occurs two-fold more from the H2 *MAPT* haplotype, which is associated with protection in neurodegenerative disorders. Therefore, hnRNP F and hnRNP Q may play a role in modulating *MAPT* neurodegenerative disease-associated susceptibility.

## Additional file

Additional file 1: Supplementary Tables and Figures. (PPTX 6588 kb)

## Abbreviations

BAC: Bacterial artificial chromosome
CBD: Corticobasal degeneration
EMSA: Electrophoretic mobility shift assay
GWAS: Genome wide association studies
LD: Linkage disequilibrium
*MAPT*: Microtubule associated protein tau
PAC: P1-derived artificial chromosome
PD: Parkinson’s disease
PSP: Progressive supranuclear palsy
SNP: Single nucleotide polymorphisms

## References

[1] Abecasis GR, Auton A, Brooks LD, DePristo MA, Durbin RM, Handsaker RE, Kang HM, Marth GT, McVean GA, Consortium GP (2012). An integrated map of genetic variation from 1,092 human genomes. Nature.

[2] Grant SF, Hakonarson H (2008). Microarray technology and applications in the arena of genome-wide association. Clin Chem.

[3] The International HapMap Consortium (2003). The international HapMap project. Nature.

[4] Lander ES, Linton LM, Birren B, Nusbaum C, Zody MC, Baldwin J, Devon K, Dewar K, Doyle M, FitzHugh W (2001). Initial sequencing and analysis of the human genome. Nature.

[5] Frazer KA, Murray SS, Schork NJ, Topol EJ (2009). Human genetic variation and its contribution to complex traits. Nat Rev Genet.

[6] Abecasis GR, Altshuler D, Auton A, Brooks LD, Durbin RM, Gibbs RA, Hurles ME, McVean GA, Consortium GP (2010). A map of human genome variation from population-scale sequencing. Nature.

[7] Schaub MA, Boyle AP, Kundaje A, Batzoglou S, Snyder M (2012). Linking disease associations with regulatory information in the human genome. Genome Res.

[8] Freedman ML, Monteiro AN, Gayther SA, Coetzee GA, Risch A, Plass C, Casey G, De Biasi M, Carlson C, Duggan D (2011). Principles for the post-GWAS functional characterization of cancer risk loci. Nat Genet.

[9] Cowper-Sal lari R, Zhang X, Wright JB, Bailey SD, Cole MD, Eeckhoute J, Moore JH, Lupien M (2012). Breast cancer risk-associated SNPs modulate the affinity of chromatin for FOXA1 and alter gene expression. Nat Genet.

[10] Pomerantz MM, Ahmadiyeh N, Jia L, Herman P, Verzi MP, Doddapaneni H, Beckwith CA, Chan JA, Hills A, Davis M (2009). The 8q24 cancer risk variant rs6983267 shows long-range interaction with MYC in colorectal cancer. Nat Genet.

[11] Maurano MT, Humbert R, Rynes E, Thurman RE, Haugen E, Wang H, Reynolds AP, Sandstrom R, Qu H, Brody J (2012). Systematic localization of common disease-associated variation in regulatory DNA. Science.

[12] Riaz M, Berns EM, Sieuwerts AM, Ruigrok-Ritstier K, de Weerd V, Groenewoud A, Uitterlinden AG, Look MP, Klijn JG, Sleijfer S (2012). Correlation of breast cancer susceptibility loci with patient characteristics, metastasis-free survival, and mRNA expression of the nearest genes. Breast Cancer Res Treat.

[13] Hoglinger GU, Melhem NM, Dickson DW, Sleiman PM, Wang LS, Klei L, Rademakers R, de Silva R, Litvan I, Riley DE (2011). Identification of common variants influencing risk of the tauopathy progressive supranuclear palsy. Nat Genet.

[14] Kouri N, Ross OA, Dombroski B, Younkin CS, Serie DJ, Soto-Ortolaza A, Baker M, Finch NC, Yoon H, Kim J (2015). Genome-wide association study of corticobasal degeneration identifies risk variants shared with progressive supranuclear palsy. Nat Commun.

[15] Nalls MA, Pankratz N, Lill CM, Do CB, Hernandez DG, Saad M, DeStefano AL, Kara E, Bras J, Sharma M (2014). Large-scale meta-analysis of genome-wide association data identifies six new risk loci for Parkinson's disease. Nat Genet.

[16] Simon-Sanchez J, Schulte C, Bras JM, Sharma M, Gibbs JR, Berg D, Paisan-Ruiz C, Lichtner P, Scholz SW, Hernandez DG (2009). Genome-wide association study reveals genetic risk underlying Parkinson's disease. Nat Genet.

[17] Stefansson H, Helgason A, Thorleifsson G, Steinthorsdottir V, Masson G, Barnard J, Baker A, Jonasdottir A, Ingason A, Gudnadottir VG (2005). A common inversion under selection in Europeans. Nat Genet.

[18] Caffrey TM, Joachim C, Paracchini S, Esiri MM, Wade-Martins R (2006). Haplotype-specific expression of exon 10 at the human *MAPT* locus. Hum Mol Genet.

[19] Caffrey TM, Joachim C, Wade-Martins R (2008). Haplotype-specific expression of the N-terminal exons 2 and 3 at the human *MAPT* locus. Neurobiol Aging.

[20] Caffrey TM, Wade-Martins R (2007). Functional *MAPT* haplotypes: bridging the gap between genotype and neuropathology. Neurobiol Dis.

[21] Spillantini MG, Murrell JR, Goedert M, Farlow MR, Klug A, Ghetti B (1998). Mutation in the tau gene in familial multiple system tauopathy with presenile dementia. Proc Natl Acad Sci U S A.

[22] Hutton M, Lendon CL, Rizzu P, Baker M, Froelich S, Houlden H, Pickering-Brown S, Chakraverty S, Isaacs A, Grover A (1998). Association of missense and 5′-splice-site mutations in tau with the inherited dementia FTDP-17. Nature.

[23] Beevers JE, Lai MC, Collins E, Booth HDE, Zambon F, Parkkinen L, Vowles J, Cowley SA, Wade-Martins R, Caffrey TM. MAPT Genetic Variation and Neuronal Maturity Alter Isoform Expression Affecting Axonal Transport in iPSC-Derived Dopamine Neurons. Stem cell reports. 2017;9:587-599. 10.1016/j.stemcr.2017.06.005 PMC5549835 28689993

[24] Liu C, Song X, Nisbet R, Gotz J (2016). Co-immunoprecipitation with tau Isoform-specific antibodies reveals distinct protein interactions and highlights a putative role for 2N tau in disease. J Biol Chem.

[25] Zhong Q, Congdon EE, Nagaraja HN, Kuret J (2012). Tau isoform composition influences rate and extent of filament formation. J Biol Chem.

[26] Peruzzi PP, Lawler SE, Senior SL, Dmitrieva N, Edser PA, Gianni D, Chiocca EA, Wade-Martins R (2009). Physiological transgene regulation and functional complementation of a neurological disease gene deficiency in neurons. Mol Ther.

[27] Wobst HJ, Denk F, Oliver PL, Livieratos A, Taylor TN, Knudsen MH, Bengoa-Vergniory N, Bannerman D, Wade-Martins R (2017). Increased 4R tau expression and behavioural changes in a novel *MAPT*-N296H genomic mouse model of tauopathy. Sci Rep.

[28] Wang J, Sarov M, Rientjes J, Fu J, Hollak H, Kranz H, Xie W, Stewart AF, Zhang Y (2006). An improved recombineering approach by adding RecA to lambda red recombination. Mol Biotechnol.

[29] Livak KJ, Schmittgen TD (2001). Analysis of relative gene expression data using real-time quantitative PCR and the 2(−Delta Delta C (T)) method. Methods.

[30] Vandesompele J, De Preter K, Pattyn F, Poppe B, Van Roy N, De Paepe A, Speleman F (2002). Accurate normalization of real-time quantitative RT-PCR data by geometric averaging of multiple internal control genes. Genome Biol.

[31] Untergasser A, Cutcutache I, Koressaar T, Ye J, Faircloth BC, Remm M, Rozen SG (2012). Primer3--new capabilities and interfaces. Nucleic Acids Res.

[32] Koressaar T, Remm M (2007). Enhancements and modifications of primer design program Primer3. Bioinformatics.

[33] Schreiber E, Matthias P, Muller MM, Schaffner W (1989). Rapid detection of octamer binding proteins with ‘mini-extracts’, prepared from a small number of cells. Nucleic Acids Res.

[34] Chambers MC, Maclean B, Burke R, Amodei D, Ruderman DL, Neumann S, Gatto L, Fischer B, Pratt B, Egertson J (2012). A cross-platform toolkit for mass spectrometry and proteomics. Nat Biotechnol.

[35] Cox J, Mann M (2008). MaxQuant enables high peptide identification rates, individualized p.P.B.-range mass accuracies and proteome-wide protein quantification. Nat Biotechnol.

[36] Giulietti M, Piva F, D'Antonio M (2013). D'Onorio de Meo P, Paoletti D, Castrignanò T, D'Erchia AM, Picardi E, Zambelli F, Principato G, et al: SpliceAid-F: a database of human splicing factors and their RNA-binding sites. Nucleic Acids Res.

[37] Pittman AM, Myers AJ, Abou-Sleiman P, Fung HC, Kaleem M, Marlowe L, Duckworth J, Leung D, Williams D, Kilford L (2005). Linkage disequilibrium fine mapping and haplotype association analysis of the tau gene in progressive supranuclear palsy and corticobasal degeneration. J Med Genet.

[38] Baker M, Litvan I, Houlden H, Adamson J, Dickson D, Perez-Tur J, Hardy J, Lynch T, Bigio E, Hutton M (1999). Association of an extended haplotype in the tau gene with progressive supranuclear palsy. Hum Mol Genet.

[39] Hecht M, Bromberg Y, Rost B (2015). Better prediction of functional effects for sequence variants. BMC Genomics.

[40] Alegre-Abarrategui J, Christian H, Lufino MM, Mutihac R, Venda LL, Ansorge O, Wade-Martins R (2009). LRRK2 regulates autophagic activity and localizes to specific membrane microdomains in a novel human genomic reporter cellular model. Hum Mol Genet.

[41] Lufino MM, Silva AM, Németh AH, Alegre-Abarrategui J, Russell AJ, Wade-Martins R (2013). A GAA repeat expansion reporter model of Friedreich's ataxia recapitulates the genomic context and allows rapid screening of therapeutic compounds. Hum Mol Genet.

[42] Desmet FO, Hamroun D, Lalande M, Collod-Beroud G, Claustres M, Beroud C (2009). Human splicing finder: an online bioinformatics tool to predict splicing signals. Nucleic Acids Res.

[43] Arikan MC, Memmott J, Broderick JA, Lafyatis R, Screaton G, Stamm S, Andreadis A (2002). Modulation of the membrane-binding projection domain of tau protein: splicing regulation of exon 3. Brain Res Mol Brain Res.

[44] Hull J, Campino S, Rowlands K, Chan MS, Copley RR, Taylor MS, Rockett K, Elvidge G, Keating B, Knight J, Kwiatkowski D (2007). Identification of common genetic variation that modulates alternative splicing. PLoS Genet.

[45] Paz I, Akerman M, Dror I, Kosti I, Mandel-Gutfreund Y (2010). SFmap: a web server for motif analysis and prediction of splicing factor binding sites. Nucleic Acids Res.

[46] Smith PJ, Zhang C, Wang J, Chew SL, Zhang MQ, Krainer AR (2006). An increased specificity score matrix for the prediction of SF2/ASF-specific exonic splicing enhancers. Hum Mol Genet.

[47] Piva F, Giulietti M, Burini AB, Principato G (2012). SpliceAid 2: a database of human splicing factors expression data and RNA target motifs. Hum Mutat.

[48] Trabzuni D, Wray S, Vandrovcova J, Ramasamy A, Walker R, Smith C, Luk C, Gibbs JR, Dillman A, Hernandez DG (2012). *MAPT* expression and splicing is differentially regulated by brain region: relation to genotype and implication for tauopathies. Hum Mol Genet.

[49] Ramanouskaya TV, Grinev VV. The determinants of alternative RNA splicing in human cells. Molecular genetics and genomics: MGG 2017. doi:10.1007/s00438-017-1350-0. 10.1007/s00438-017-1350-0 28707092

[50] Fu XD, Ares M (2014). Context-dependent control of alternative splicing by RNA-binding proteins. Nat Rev Genet.

[51] Cooper TA (2005). Use of minigene systems to dissect alternative splicing elements. Methods.

[52] Degner JF, Pai AA, Pique-Regi R, Veyrieras JB, Gaffney DJ, Pickrell JK, De Leon S, Michelini K, Lewellen N, Crawford GE (2012). DNase I sensitivity QTLs are a major determinant of human expression variation. Nature.

[53] Kwan T, Benovoy D, Dias C, Gurd S, Provencher C, Beaulieu P, Hudson TJ, Sladek R, Majewski J (2008). Genome-wide analysis of transcript isoform variation in humans. Nat Genet.

[54] Rimoldi V, Solda G, Asselta R, Spena S, Stuani C, Buratti E, Duga S (2013). Dual role of G-runs and hnRNP F in the regulation of a mutation-activated pseudoexon in the fibrinogen gamma-chain transcript. PLoS One.

[55] Tacheny A, Dieu M, Arnould T, Renard P (2013). Mass spectrometry-based identification of proteins interacting with nucleic acids. J Proteome.

[56] Nahnsen S, Bielow C, Reinert K, Kohlbacher O (2013). Tools for label-free peptide quantification. Mol Cell Proteomics.

[57] Huelga SC, Vu AQ, Arnold JD, Liang TY, Liu PP, Yan BY, Donohue JP, Shiue L, Hoon S, Brenner S (2012). Integrative genome-wide analysis reveals cooperative regulation of alternative splicing by hnRNP proteins. Cell Rep.

[58] Wang E, Aslanzadeh V, Papa F, Zhu H, de la Grange P, Cambi F: Global profiling of alternative splicing events and gene expression regulated by hnRNPH/F. PLoS One 2012, 7:e51266. 10.1371/journal.pone.0051266 PMC3524136 23284676

[59] Andreadis A, Broderick JA, Kosik KS (1995). Relative exon affinities and suboptimal splice site signals lead to non-equivalence of two cassette exons. Nucleic Acids Res.

[60] Brandt R, Leger J, Lee G (1995). Interaction of tau with the neural plasma membrane mediated by tau's amino-terminal projection domain. J Cell Biol.

[61] Magnani E, Fan J, Gasparini L, Golding M, Williams M, Schiavo G, Goedert M, Amos LA, Spillantini MG (2007). Interaction of tau protein with the dynactin complex. EMBO J.

[62] Lee G, Newman ST, Gard DL, Band H, Panchamoorthy G (1998). Tau interacts with src-family non-receptor tyrosine kinases. J Cell Sci.

[63] Shahani N, Brandt R (2002). Functions and malfunctions of the tau proteins. Cell Mol Life Sci.

[64] Kempf M, Clement A, Faissner A, Lee G, Brandt R (1996). Tau binds to the distal axon early in development of polarity in a microtubule- and microfilament-dependent manner. J Neurosci.

[65] Cunningham CC, Leclerc N, Flanagan LA, Lu M, Janmey PA, Kosik KS (1997). Microtubule-associated protein 2c reorganizes both microtubules and microfilaments into distinct cytological structures in an actin-binding protein-280-deficient melanoma cell line. J Cell Biol.

[66] DiTella M, Feiguin F, Morfini G, Caceres A (1994). Microfilament-associated growth cone component depends upon tau for its intracellular localization. Cell Motil Cytoskeleton.

[67] Hwang SC, Jhon DY, Bae YS, Kim JH, Rhee SG (1996). Activation of phospholipase C-gamma by the concerted action of tau proteins and arachidonic acid. J Biol Chem.

[68] Jenkins SM, Johnson GV (1998). Tau complexes with phospholipase C-gamma in situ. Neuroreport.

[69] Dixit R, Ross JL, Goldman YE, Holzbaur ELF (2008). Differential regulation of Dynein and Kinesin motor proteins by tau. Science.

[70] Saha AR, Hill J, Utton MA, Asuni AA, Ackerley S, Grierson AJ, Miller CC, Davies AM, Buchman VL, Anderton BH, Hanger DP (2004). Parkinson's disease alpha-synuclein mutations exhibit defective axonal transport in cultured neurons. J Cell Sci.

[71] Panicker N, Saminathan H, Jin H, Neal M, Harischandra DS, Gordon R, Kanthasamy K, Lawana V, Sarkar S, Luo J (2015). Fyn Kinase regulates Microglial Neuroinflammatory responses in cell culture and animal models of Parkinson's disease. J Neurosci.

[72] Sandebring A, Dehvari N, Perez-Manso M, Thomas KJ, Karpilovski E, Cookson MR, Cowburn RF, Cedazo-Minguez A (2009). Parkin deficiency disrupts calcium homeostasis by modulating phospholipase C signalling. FEBS J.

[73] Feany MB, Ksiezak-Reding H, Liu WK, Vincent I, Yen SH, Dickson DW (1995). Epitope expression and hyperphosphorylation of tau protein in corticobasal degeneration: differentiation from progressive supranuclear palsy. Acta Neuropathol (Berl).

[74] Arai T, Ikeda K, Akiyama H, Shikamoto Y, Tsuchiya K, Yagishita S, Beach T, Rogers J, Schwab C, McGeer PL (2001). Distinct isoforms of tau aggregated in neurons and glial cells in brains of patients with Pick's disease, corticobasal degeneration and progressive supranuclear palsy. Acta Neuropathol (Berl).

