# Supplementary material for: Haplotype-specific MAPT exon 3 expression regulated by common intronic polymorphisms associated with Parkinsonian disorders
Source: Mol Neurodegener. 2017 Oct 30;12:79. doi: 10.1186/s13024-017-0224-6 (PMC5663040; doi:10.1186/s13024-017-0224-6)
Supplement: Additional file 1: — Supplementary Tables and Figures. (PPTX 6588 kb) [file 13024_2017_224_MOESM1_ESM.pptx]

## Slide 1
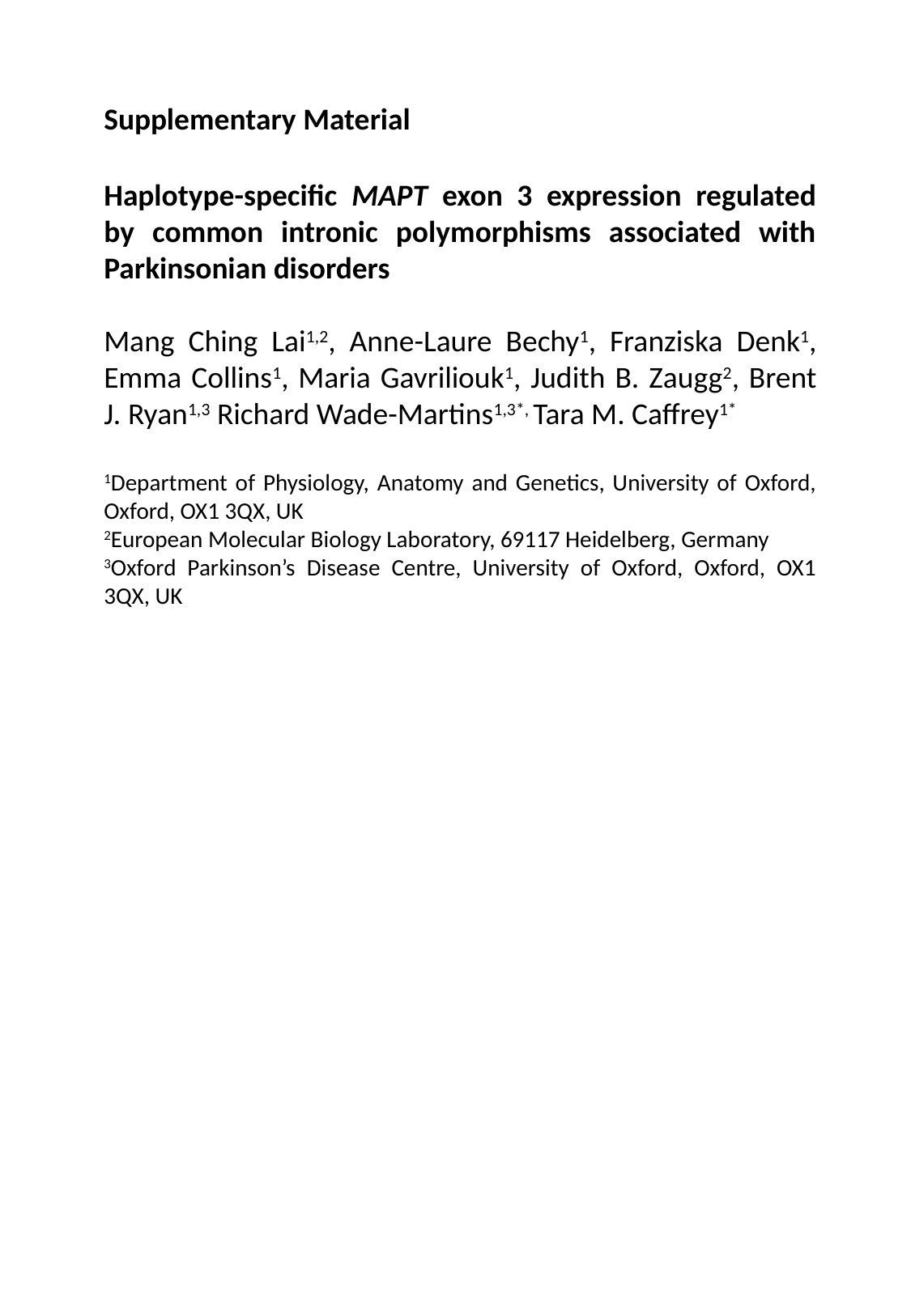

Supplementary Material
Haplotype-specific MAPT exon 3 expression regulated by common intronic polymorphisms associated with Parkinsonian disorders
Mang Ching Lai1,2, Anne-Laure Bechy1, Franziska Denk1, Emma Collins1, Maria Gavriliouk1, Judith B. Zaugg2, Brent J. Ryan1,3 Richard Wade-Martins1,3*, Tara M. Caffrey1*
1Department of Physiology, Anatomy and Genetics, University of Oxford, Oxford, OX1 3QX, UK
2European Molecular Biology Laboratory, 69117 Heidelberg, Germany
3Oxford Parkinson’s Disease Centre, University of Oxford, Oxford, OX1 3QX, UK

## Slide 2
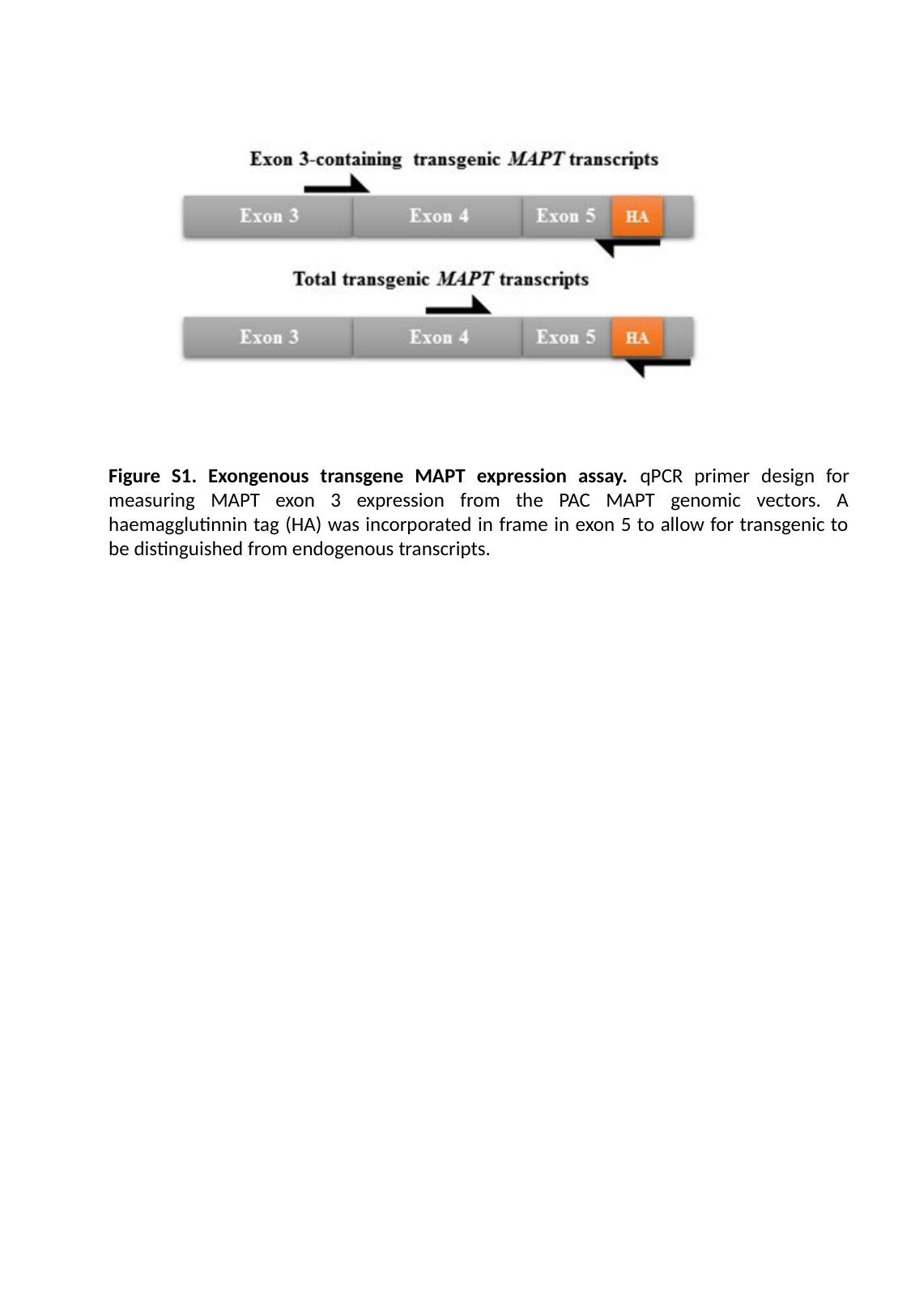

Figure S1. Exongenous transgene MAPT expression assay. qPCR primer design for measuring MAPT exon 3 expression from the PAC MAPT genomic vectors. A haemagglutinnin tag (HA) was incorporated in frame in exon 5 to allow for transgenic to be distinguished from endogenous transcripts.

## Slide 3
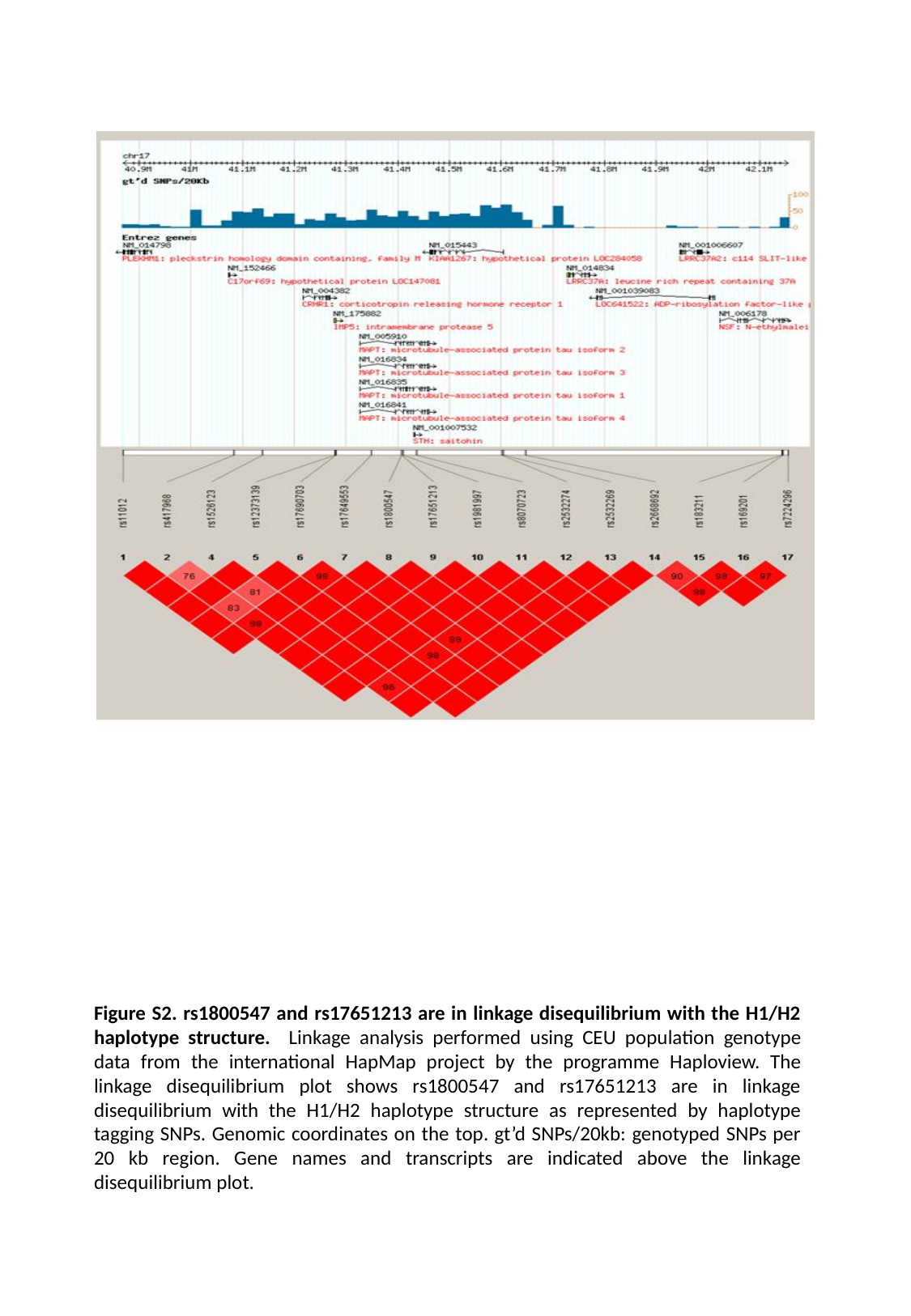

Figure S2. rs1800547 and rs17651213 are in linkage disequilibrium with the H1/H2 haplotype structure. Linkage analysis performed using CEU population genotype data from the international HapMap project by the programme Haploview. The linkage disequilibrium plot shows rs1800547 and rs17651213 are in linkage disequilibrium with the H1/H2 haplotype structure as represented by haplotype tagging SNPs. Genomic coordinates on the top. gt’d SNPs/20kb: genotyped SNPs per 20 kb region. Gene names and transcripts are indicated above the linkage disequilibrium plot.

## Slide 4
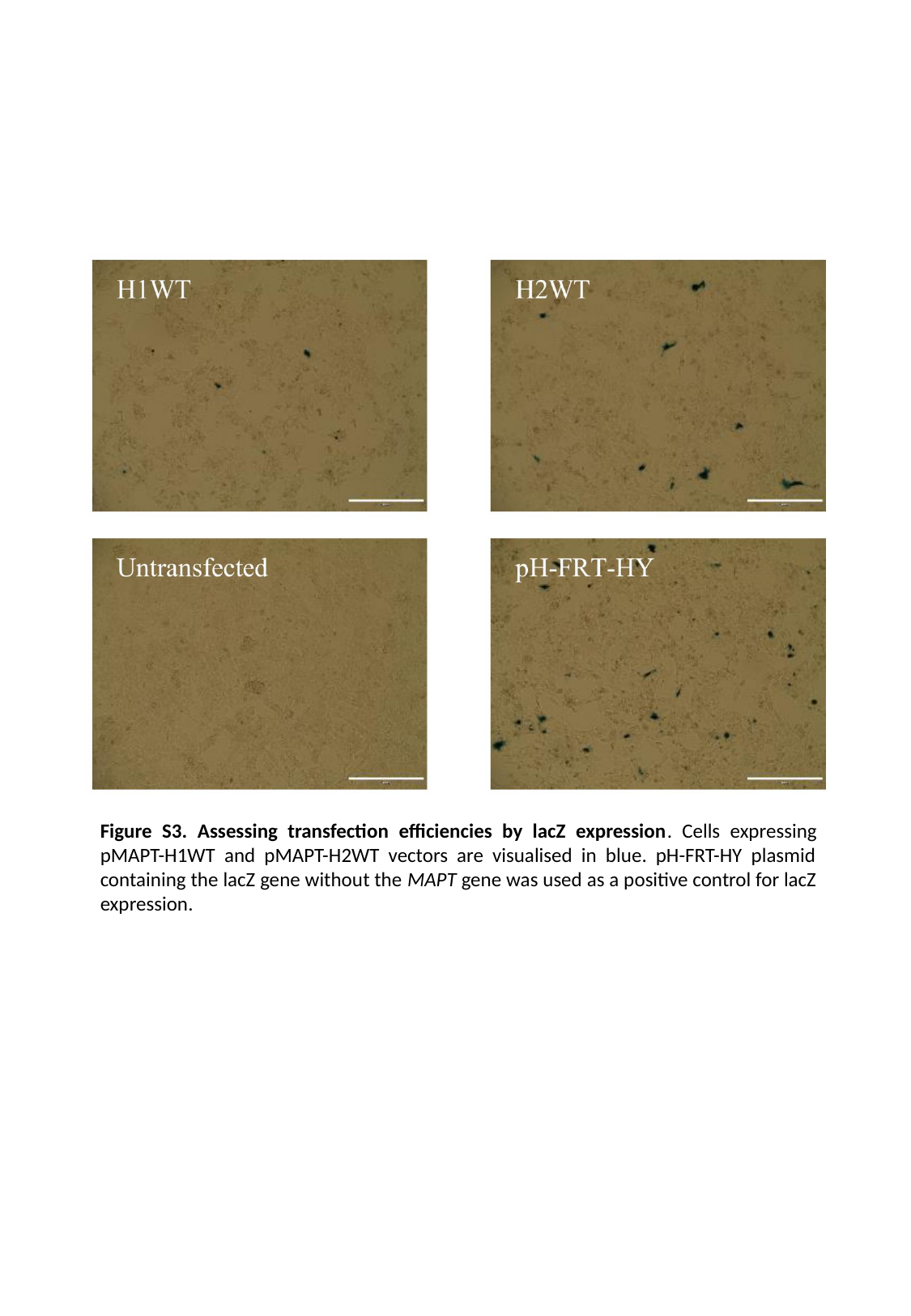

Figure S3. Assessing transfection efficiencies by lacZ expression. Cells expressing pMAPT-H1WT and pMAPT-H2WT vectors are visualised in blue. pH-FRT-HY plasmid containing the lacZ gene without the MAPT gene was used as a positive control for lacZ expression.

## Slide 5
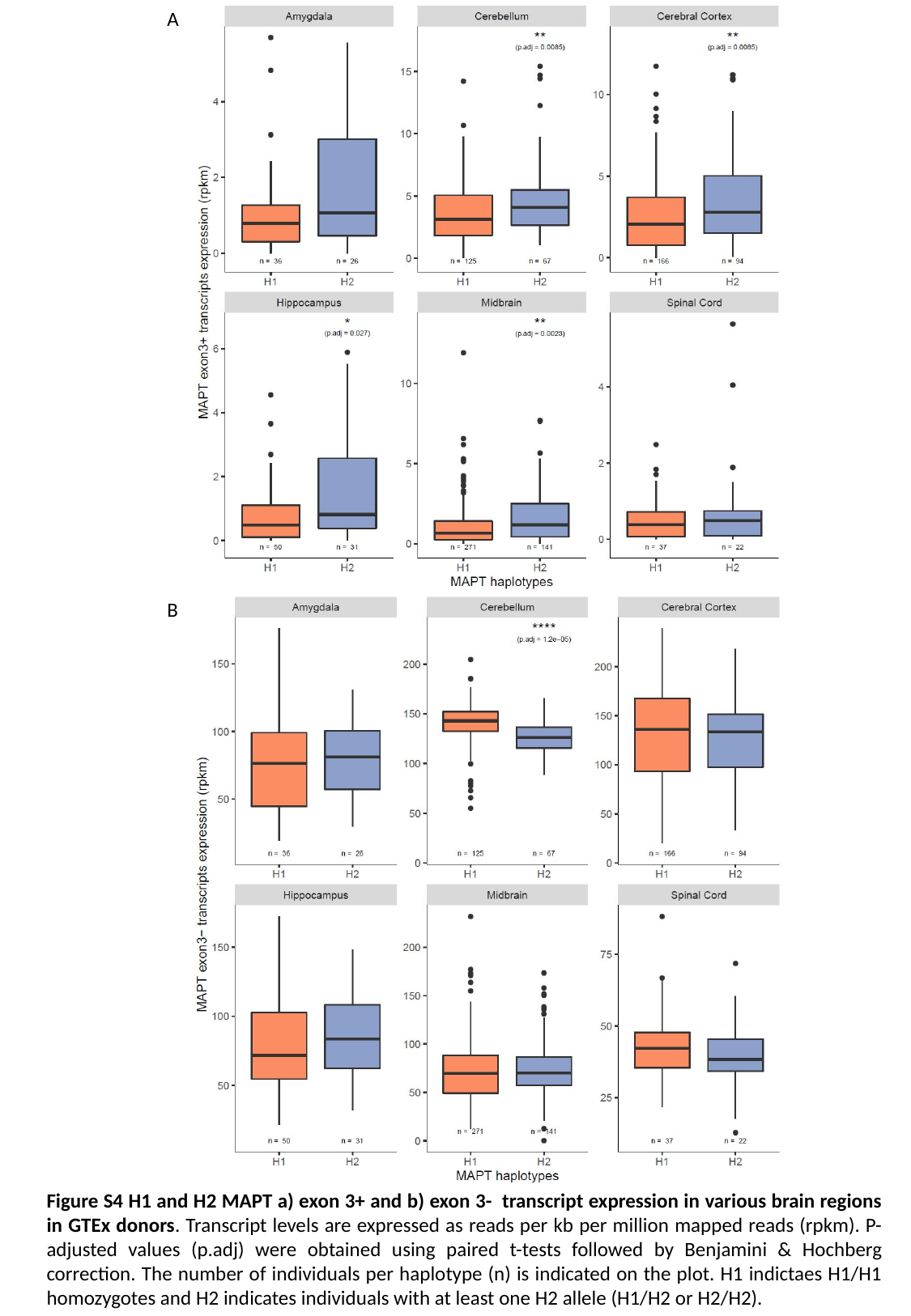

A
B
Figure S4 H1 and H2 MAPT a) exon 3+ and b) exon 3- transcript expression in various brain regions in GTEx donors. Transcript levels are expressed as reads per kb per million mapped reads (rpkm). P-adjusted values (p.adj) were obtained using paired t-tests followed by Benjamini & Hochberg correction. The number of individuals per haplotype (n) is indicated on the plot. H1 indictaes H1/H1 homozygotes and H2 indicates individuals with at least one H2 allele (H1/H2 or H2/H2).

## Slide 6
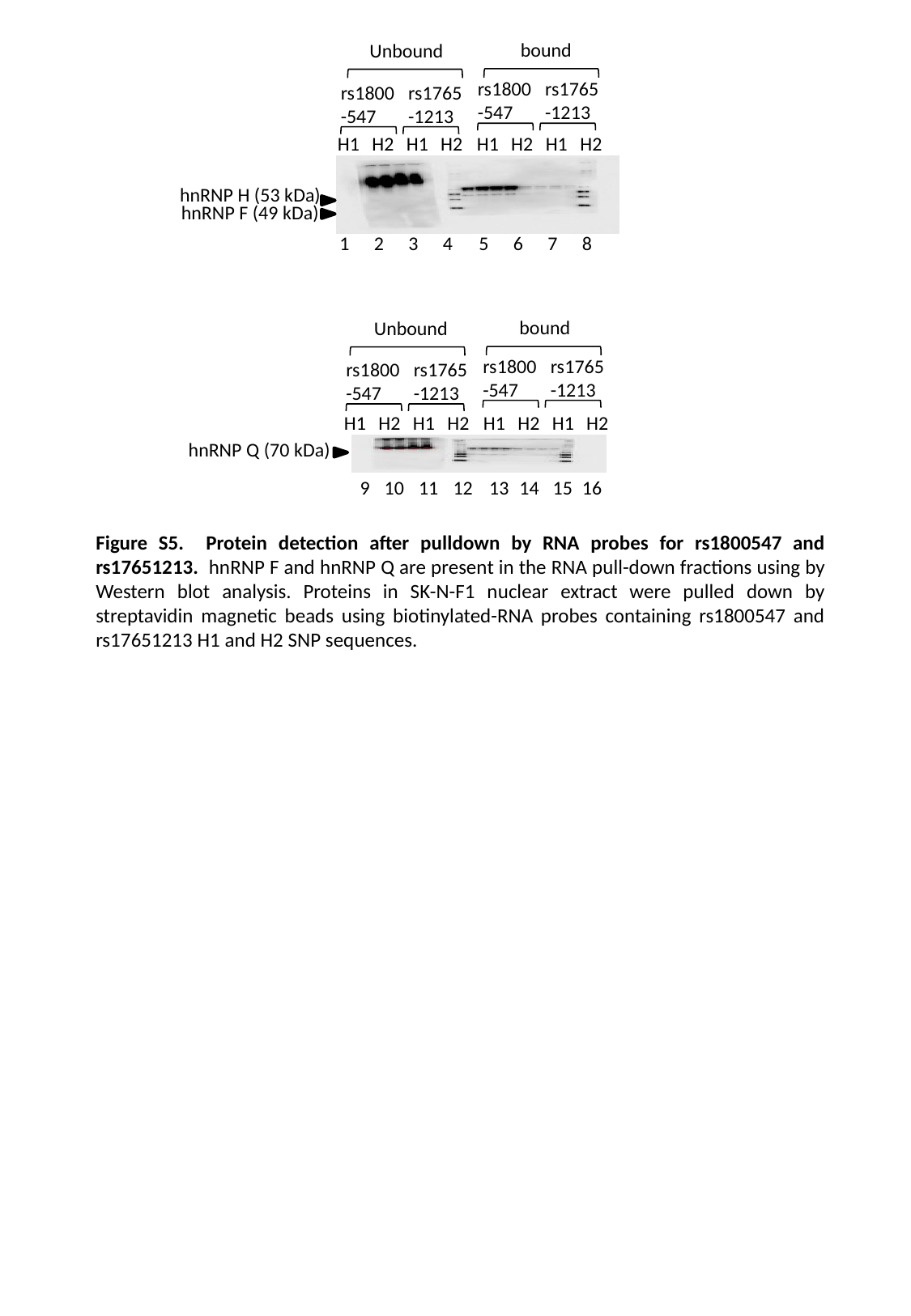

bound
Unbound
rs1765
-1213
rs1800
-547
rs1765
-1213
rs1800
-547
H1
H2
H1
H2
H1
H2
H1
H2
hnRNP H (53 kDa)
hnRNP F (49 kDa)
1
2
3
4
5
6
7
8
bound
Unbound
rs1765
-1213
rs1800
-547
rs1765
-1213
rs1800
-547
H1
H2
H1
H2
H1
H2
H1
H2
hnRNP Q (70 kDa)
9
10
11
12
13
14
15
16
Figure S5. Protein detection after pulldown by RNA probes for rs1800547 and rs17651213. hnRNP F and hnRNP Q are present in the RNA pull-down fractions using by Western blot analysis. Proteins in SK-N-F1 nuclear extract were pulled down by streptavidin magnetic beads using biotinylated-RNA probes containing rs1800547 and rs17651213 H1 and H2 SNP sequences.

## Slide 7
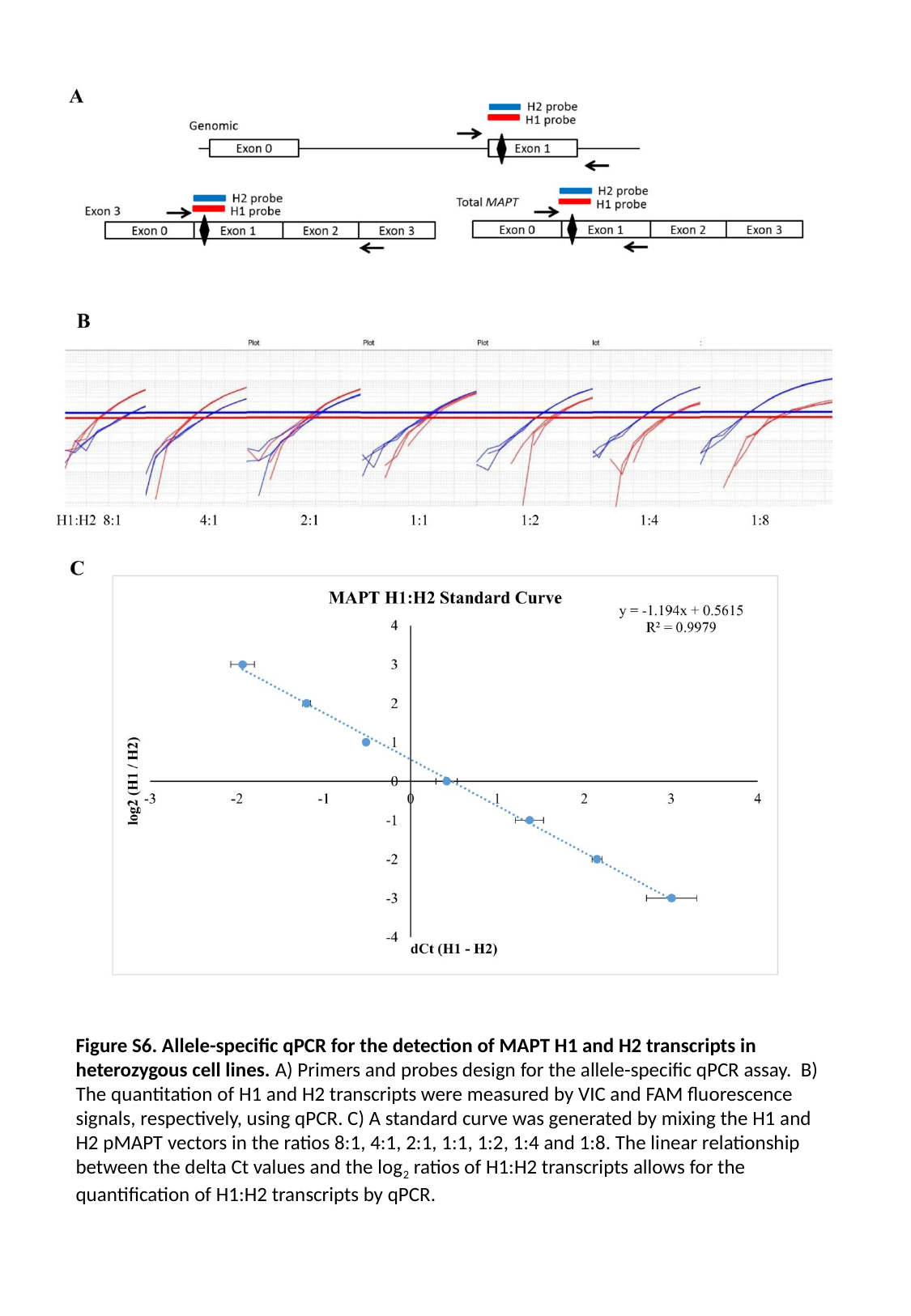

Figure S6. Allele-specific qPCR for the detection of MAPT H1 and H2 transcripts in heterozygous cell lines. A) Primers and probes design for the allele-specific qPCR assay. B) The quantitation of H1 and H2 transcripts were measured by VIC and FAM fluorescence signals, respectively, using qPCR. C) A standard curve was generated by mixing the H1 and H2 pMAPT vectors in the ratios 8:1, 4:1, 2:1, 1:1, 1:2, 1:4 and 1:8. The linear relationship between the delta Ct values and the log2 ratios of H1:H2 transcripts allows for the quantification of H1:H2 transcripts by qPCR.

## Slide 8
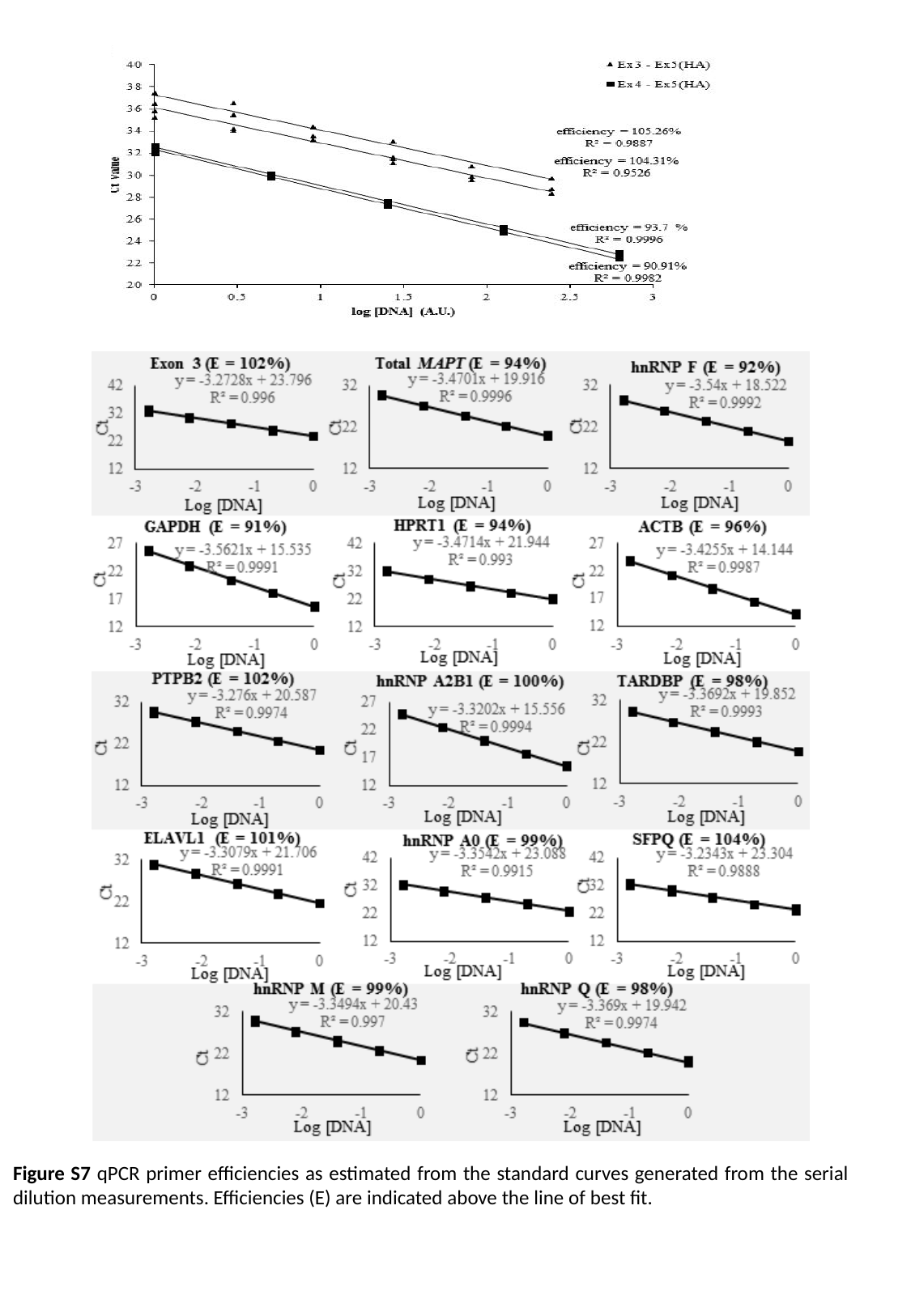

Figure S7 qPCR primer efficiencies as estimated from the standard curves generated from the serial dilution measurements. Efficiencies (E) are indicated above the line of best fit.

## Slide 9
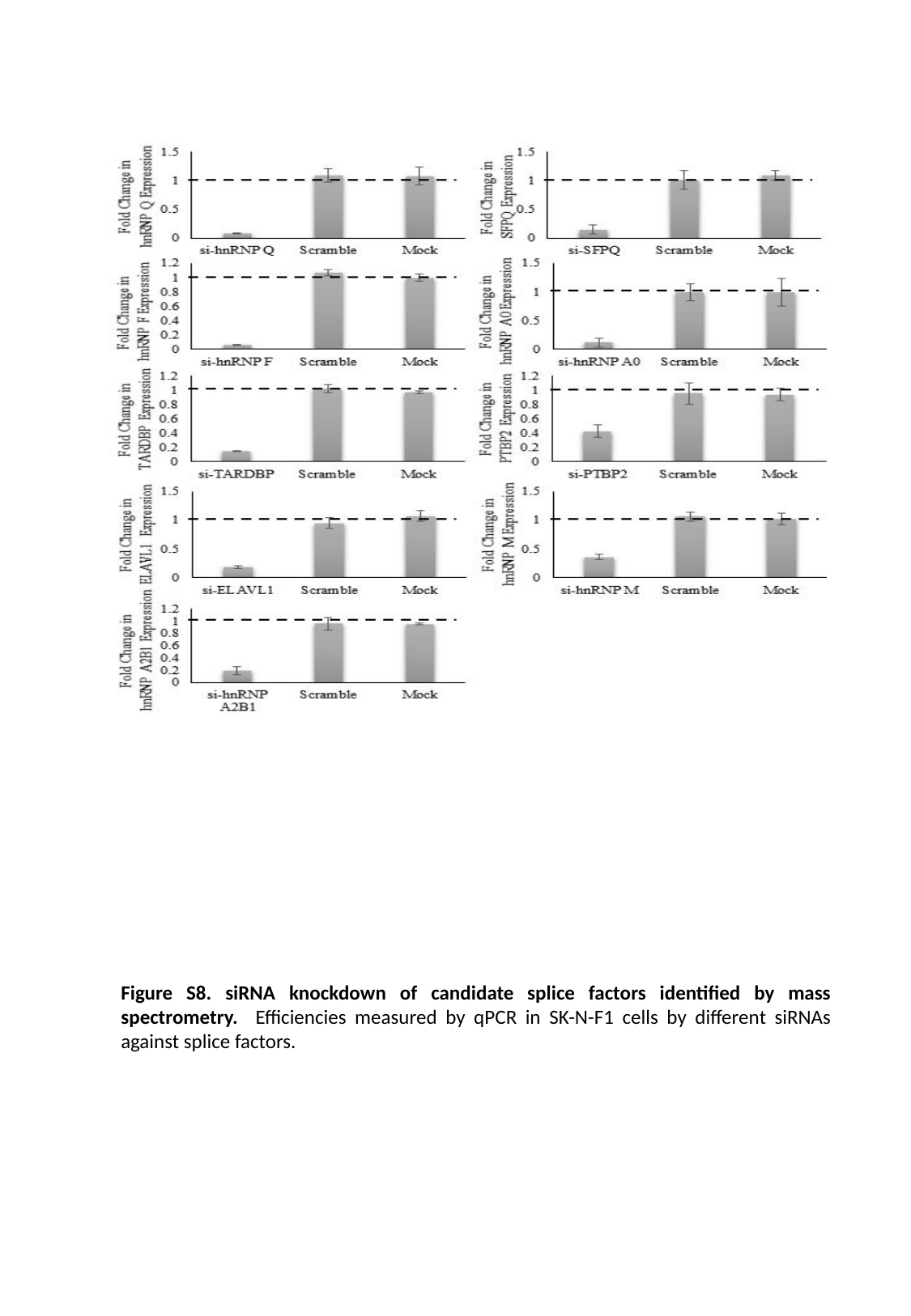

Figure S8. siRNA knockdown of candidate splice factors identified by mass spectrometry. Efficiencies measured by qPCR in SK-N-F1 cells by different siRNAs against splice factors.

## Slide 10
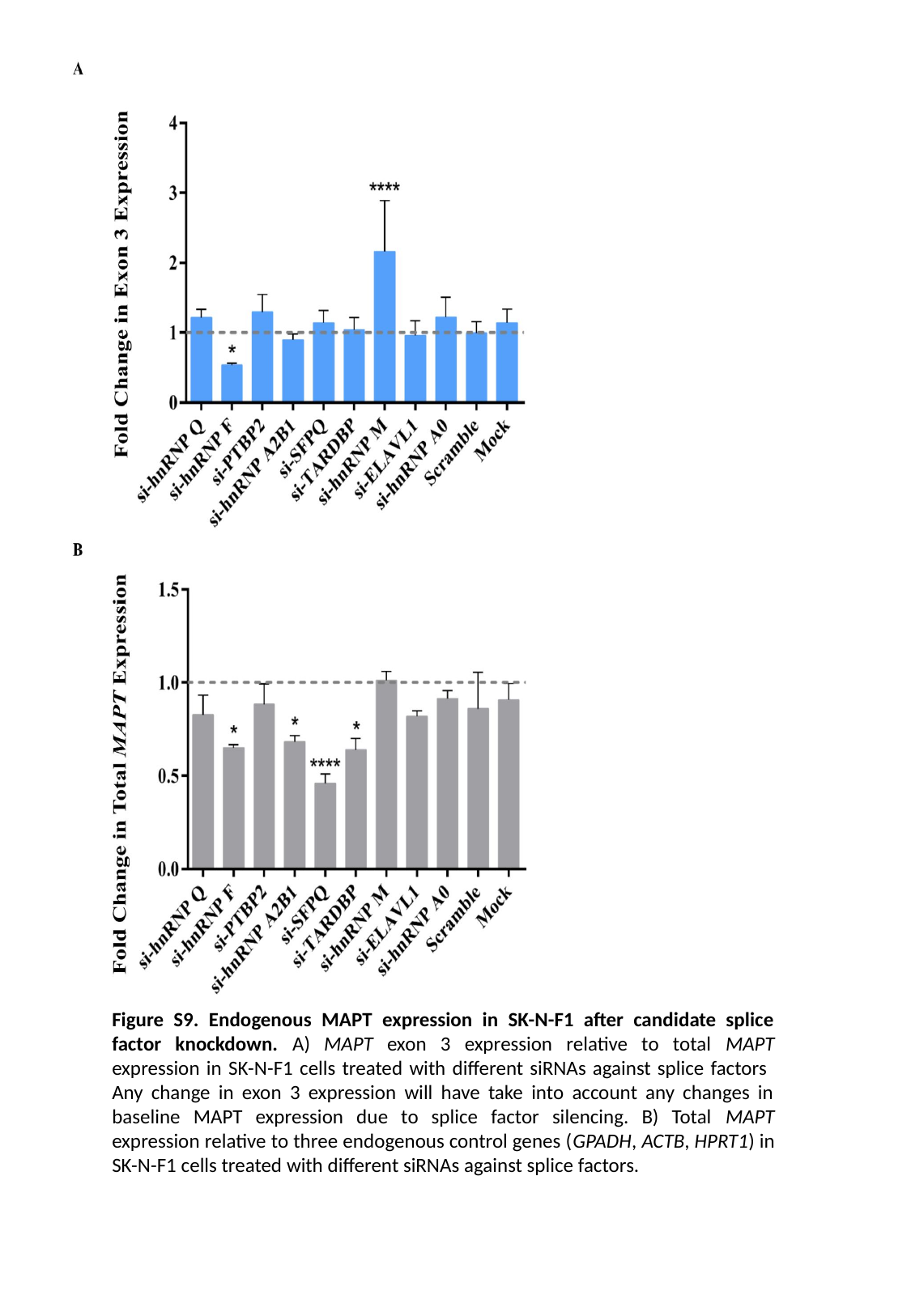

Figure S9. Endogenous MAPT expression in SK-N-F1 after candidate splice factor knockdown. A) MAPT exon 3 expression relative to total MAPT expression in SK-N-F1 cells treated with different siRNAs against splice factors Any change in exon 3 expression will have take into account any changes in baseline MAPT expression due to splice factor silencing. B) Total MAPT expression relative to three endogenous control genes (GPADH, ACTB, HPRT1) in SK-N-F1 cells treated with different siRNAs against splice factors.

## Slide 11
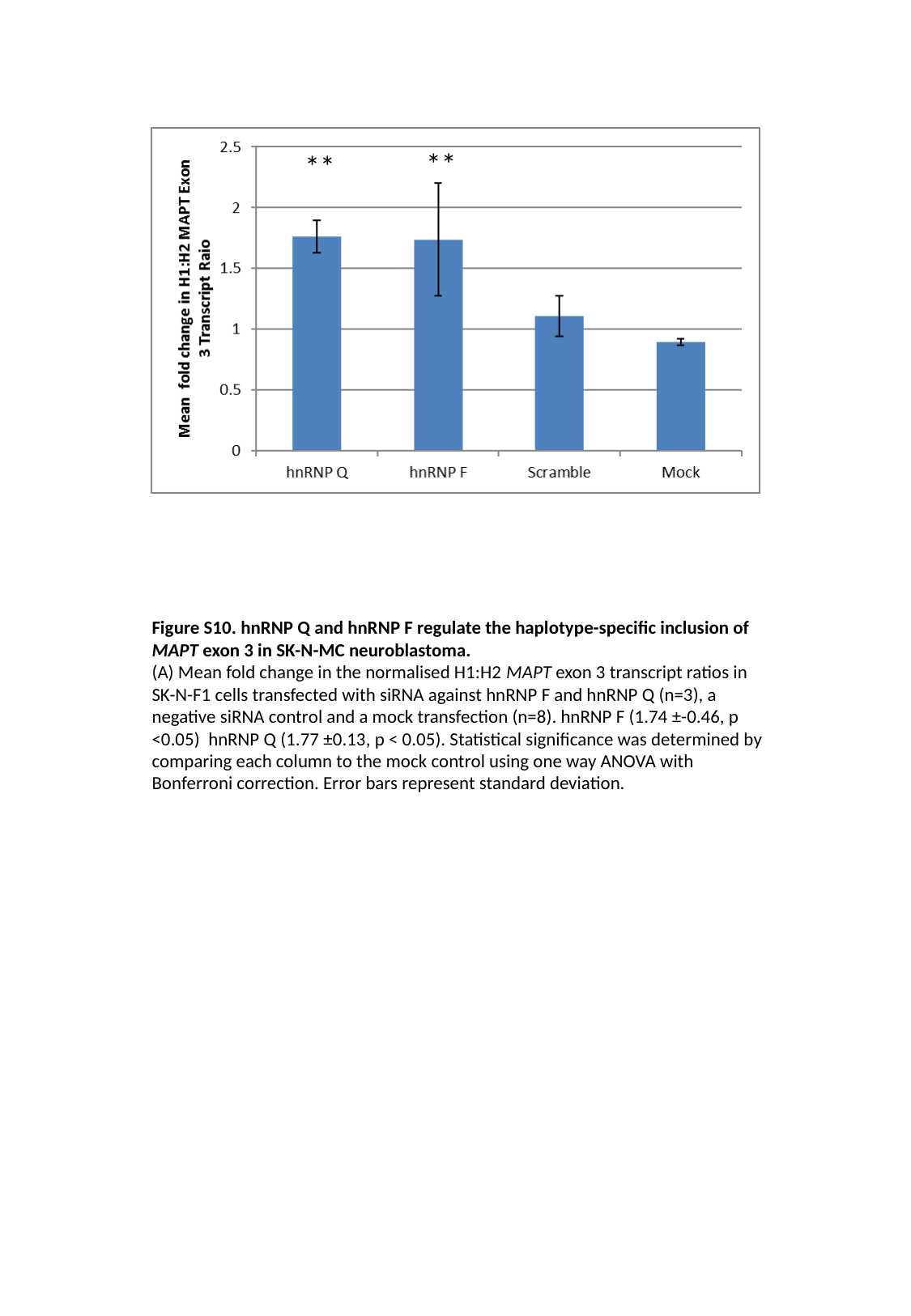

**
**
Figure S10. hnRNP Q and hnRNP F regulate the haplotype-specific inclusion of MAPT exon 3 in SK-N-MC neuroblastoma.
(A) Mean fold change in the normalised H1:H2 MAPT exon 3 transcript ratios in SK-N-F1 cells transfected with siRNA against hnRNP F and hnRNP Q (n=3), a negative siRNA control and a mock transfection (n=8). hnRNP F (1.74 ±-0.46, p <0.05) hnRNP Q (1.77 ±0.13, p < 0.05). Statistical significance was determined by comparing each column to the mock control using one way ANOVA with Bonferroni correction. Error bars represent standard deviation.

## Slide 12
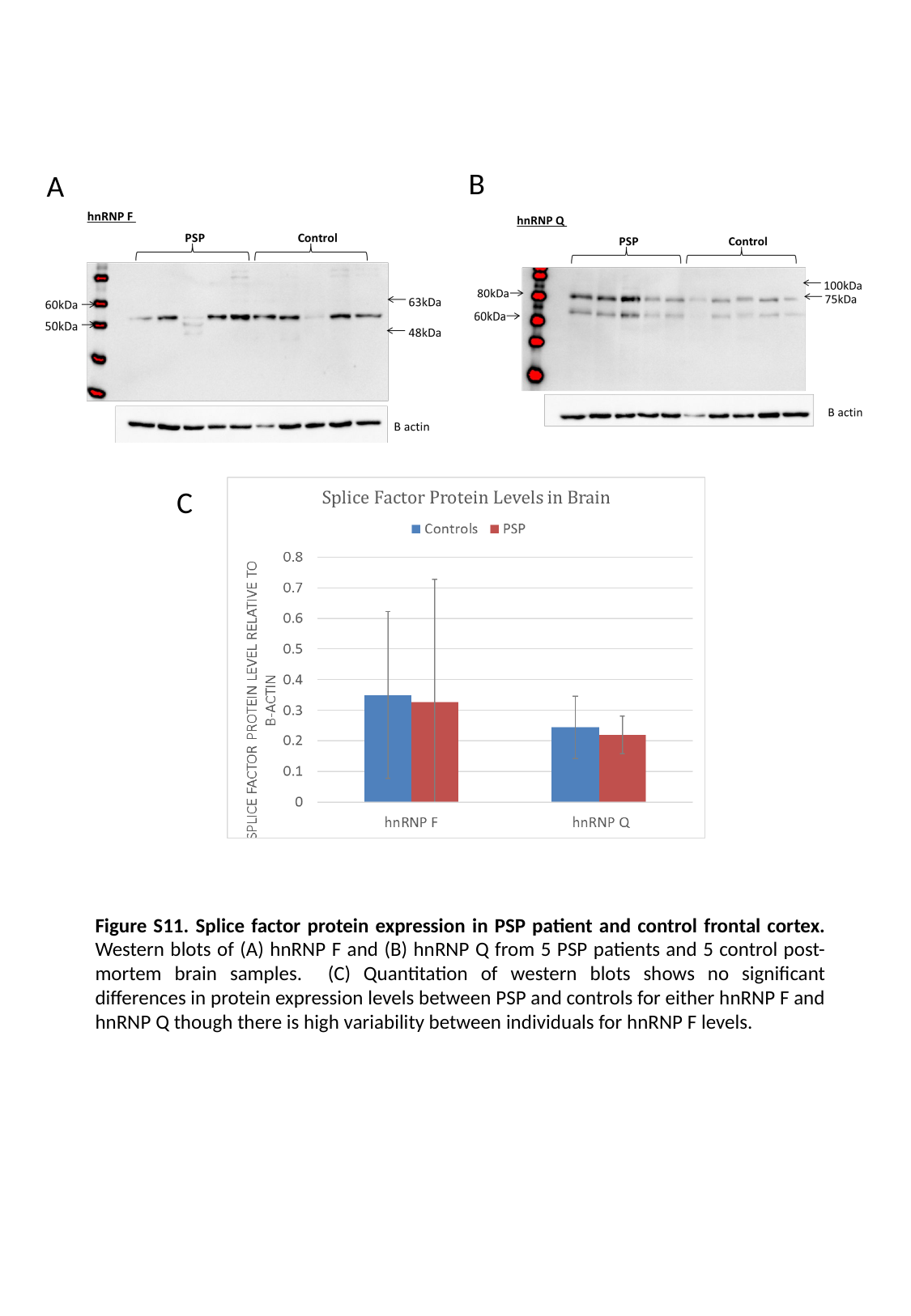

B
A
C
Figure S11. Splice factor protein expression in PSP patient and control frontal cortex. Western blots of (A) hnRNP F and (B) hnRNP Q from 5 PSP patients and 5 control post-mortem brain samples. (C) Quantitation of western blots shows no significant differences in protein expression levels between PSP and controls for either hnRNP F and hnRNP Q though there is high variability between individuals for hnRNP F levels.

## Slide 13
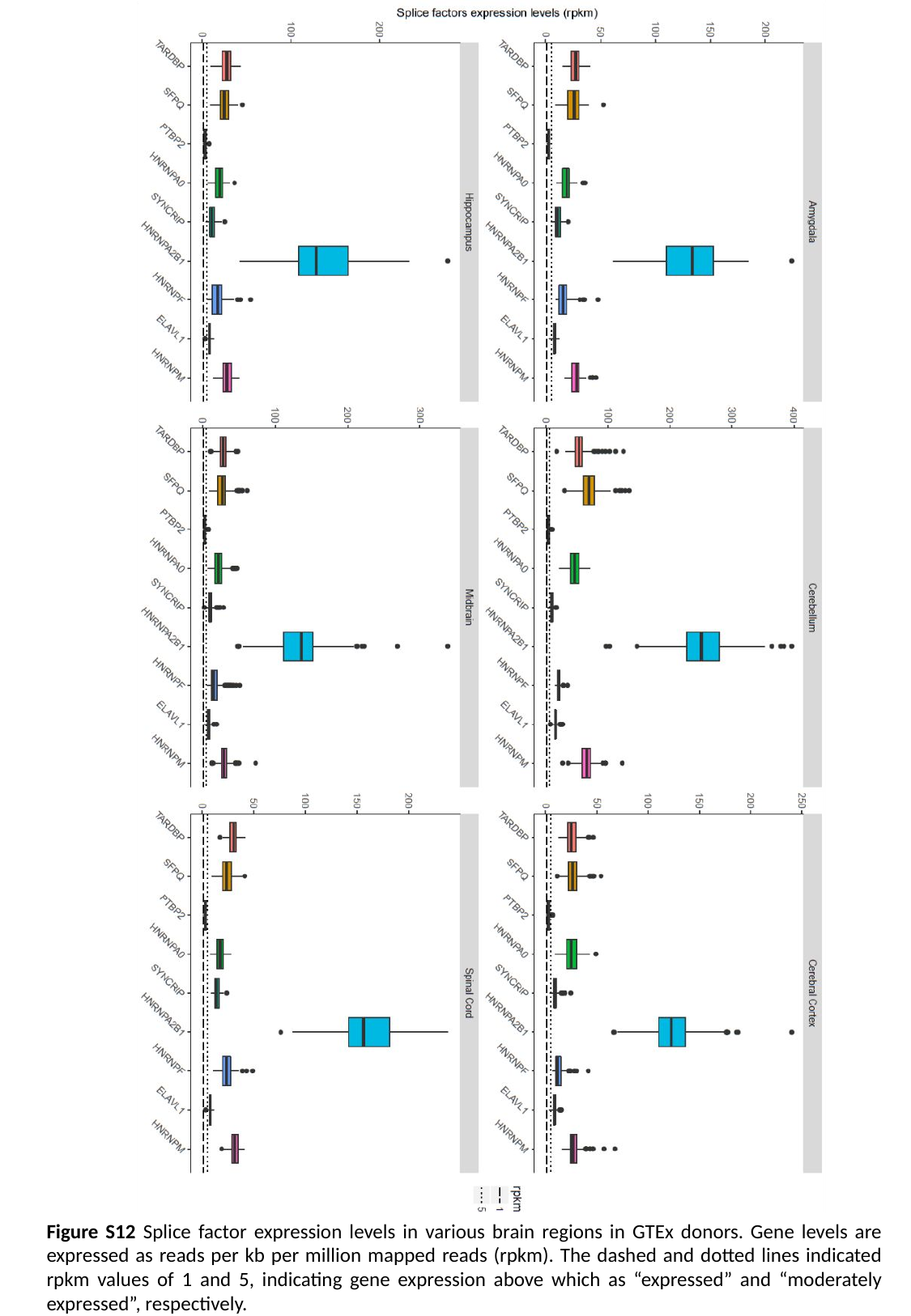

Figure S12 Splice factor expression levels in various brain regions in GTEx donors. Gene levels are expressed as reads per kb per million mapped reads (rpkm). The dashed and dotted lines indicated rpkm values of 1 and 5, indicating gene expression above which as “expressed” and “moderately expressed”, respectively.

## Slide 14
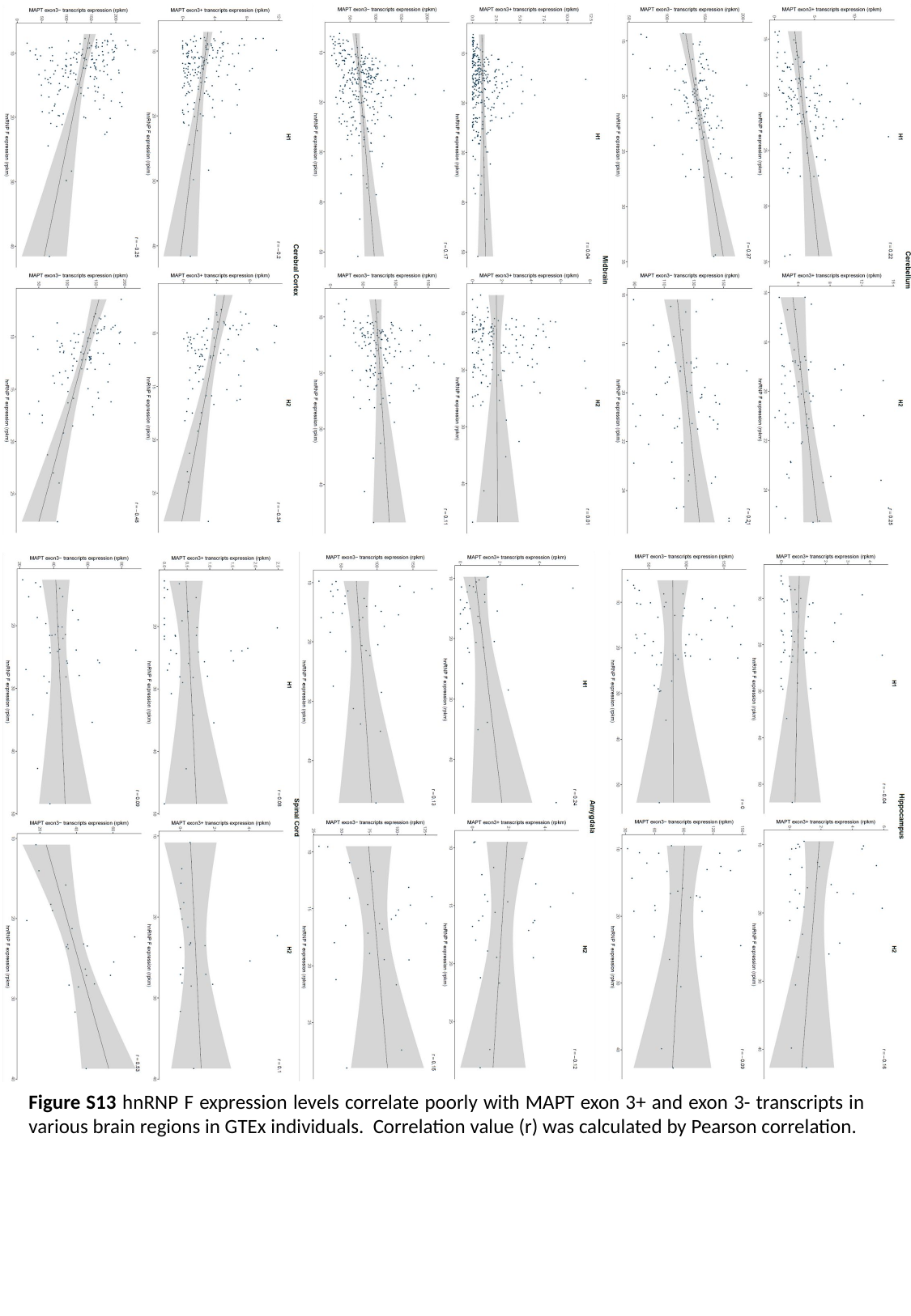

Figure S13 hnRNP F expression levels correlate poorly with MAPT exon 3+ and exon 3- transcripts in various brain regions in GTEx individuals. Correlation value (r) was calculated by Pearson correlation.

## Slide 15
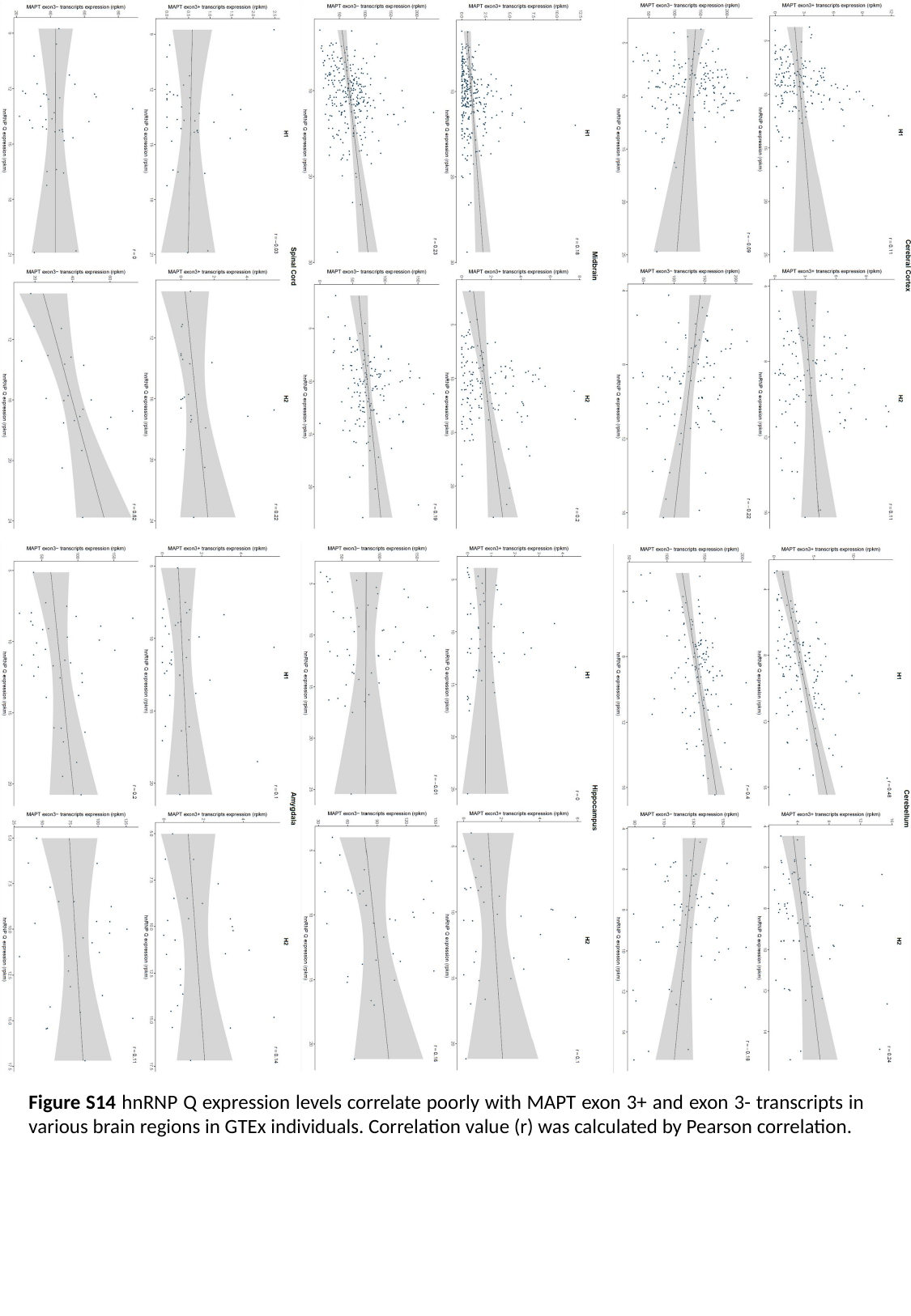

Figure S14 hnRNP Q expression levels correlate poorly with MAPT exon 3+ and exon 3- transcripts in various brain regions in GTEx individuals. Correlation value (r) was calculated by Pearson correlation.

## Slide 16
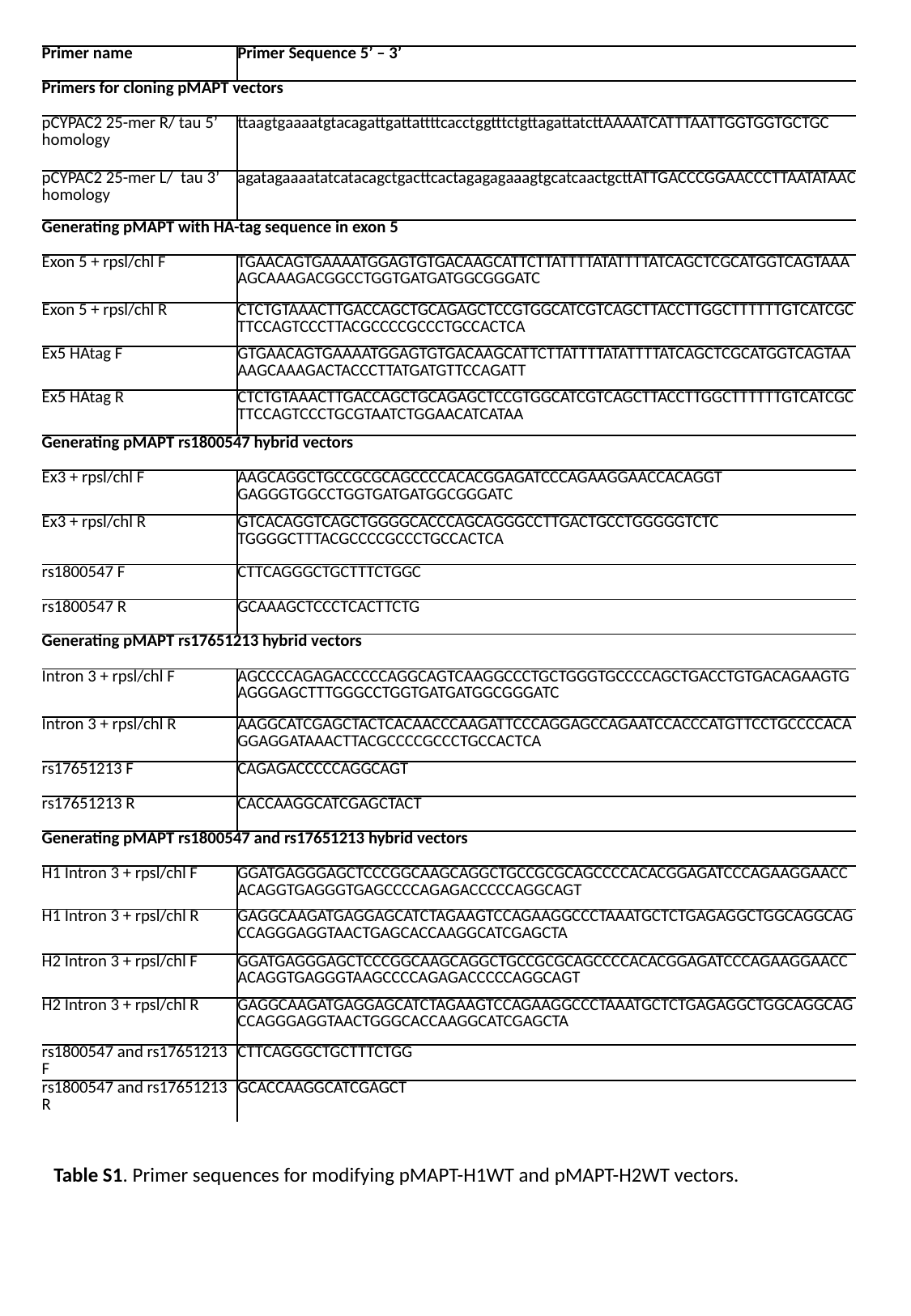

| Primer name | Primer Sequence 5’ – 3’ |
| --- | --- |
| Primers for cloning pMAPT vectors | |
| pCYPAC2 25-mer R/ tau 5’ homology | ttaagtgaaaatgtacagattgattattttcacctggtttctgttagattatcttAAAATCATTTAATTGGTGGTGCTGC |
| pCYPAC2 25-mer L/  tau 3’ homology | agatagaaaatatcatacagctgacttcactagagagaaagtgcatcaactgcttATTGACCCGGAACCCTTAATATAAC |
| Generating pMAPT with HA-tag sequence in exon 5 | |
| Exon 5 + rpsl/chl F | TGAACAGTGAAAATGGAGTGTGACAAGCATTCTTATTTTATATTTTATCAGCTCGCATGGTCAGTAAAAGCAAAGACGGCCTGGTGATGATGGCGGGATC |
| Exon 5 + rpsl/chl R | CTCTGTAAACTTGACCAGCTGCAGAGCTCCGTGGCATCGTCAGCTTACCTTGGCTTTTTTGTCATCGCTTCCAGTCCCTTACGCCCCGCCCTGCCACTCA |
| Ex5 HAtag F | GTGAACAGTGAAAATGGAGTGTGACAAGCATTCTTATTTTATATTTTATCAGCTCGCATGGTCAGTAAAAGCAAAGACTACCCTTATGATGTTCCAGATT |
| Ex5 HAtag R | CTCTGTAAACTTGACCAGCTGCAGAGCTCCGTGGCATCGTCAGCTTACCTTGGCTTTTTTGTCATCGCTTCCAGTCCCTGCGTAATCTGGAACATCATAA |
| Generating pMAPT rs1800547 hybrid vectors | |
| Ex3 + rpsl/chl F | AAGCAGGCTGCCGCGCAGCCCCACACGGAGATCCCAGAAGGAACCACAGGT GAGGGTGGCCTGGTGATGATGGCGGGATC |
| Ex3 + rpsl/chl R | GTCACAGGTCAGCTGGGGCACCCAGCAGGGCCTTGACTGCCTGGGGGTCTC TGGGGCTTTACGCCCCGCCCTGCCACTCA |
| rs1800547 F | CTTCAGGGCTGCTTTCTGGC |
| rs1800547 R | GCAAAGCTCCCTCACTTCTG |
| Generating pMAPT rs17651213 hybrid vectors | |
| Intron 3 + rpsl/chl F | AGCCCCAGAGACCCCCAGGCAGTCAAGGCCCTGCTGGGTGCCCCAGCTGACCTGTGACAGAAGTGAGGGAGCTTTGGGCCTGGTGATGATGGCGGGATC |
| Intron 3 + rpsl/chl R | AAGGCATCGAGCTACTCACAACCCAAGATTCCCAGGAGCCAGAATCCACCCATGTTCCTGCCCCACAGGAGGATAAACTTACGCCCCGCCCTGCCACTCA |
| rs17651213 F | CAGAGACCCCCAGGCAGT |
| rs17651213 R | CACCAAGGCATCGAGCTACT |
| Generating pMAPT rs1800547 and rs17651213 hybrid vectors | |
| H1 Intron 3 + rpsl/chl F | GGATGAGGGAGCTCCCGGCAAGCAGGCTGCCGCGCAGCCCCACACGGAGATCCCAGAAGGAACCACAGGTGAGGGTGAGCCCCAGAGACCCCCAGGCAGT |
| H1 Intron 3 + rpsl/chl R | GAGGCAAGATGAGGAGCATCTAGAAGTCCAGAAGGCCCTAAATGCTCTGAGAGGCTGGCAGGCAGCCAGGGAGGTAACTGAGCACCAAGGCATCGAGCTA |
| H2 Intron 3 + rpsl/chl F | GGATGAGGGAGCTCCCGGCAAGCAGGCTGCCGCGCAGCCCCACACGGAGATCCCAGAAGGAACCACAGGTGAGGGTAAGCCCCAGAGACCCCCAGGCAGT |
| H2 Intron 3 + rpsl/chl R | GAGGCAAGATGAGGAGCATCTAGAAGTCCAGAAGGCCCTAAATGCTCTGAGAGGCTGGCAGGCAGCCAGGGAGGTAACTGGGCACCAAGGCATCGAGCTA |
| rs1800547 and rs17651213 F | CTTCAGGGCTGCTTTCTGG |
| rs1800547 and rs17651213 R | GCACCAAGGCATCGAGCT |
Table S1. Primer sequences for modifying pMAPT-H1WT and pMAPT-H2WT vectors.

## Slide 17
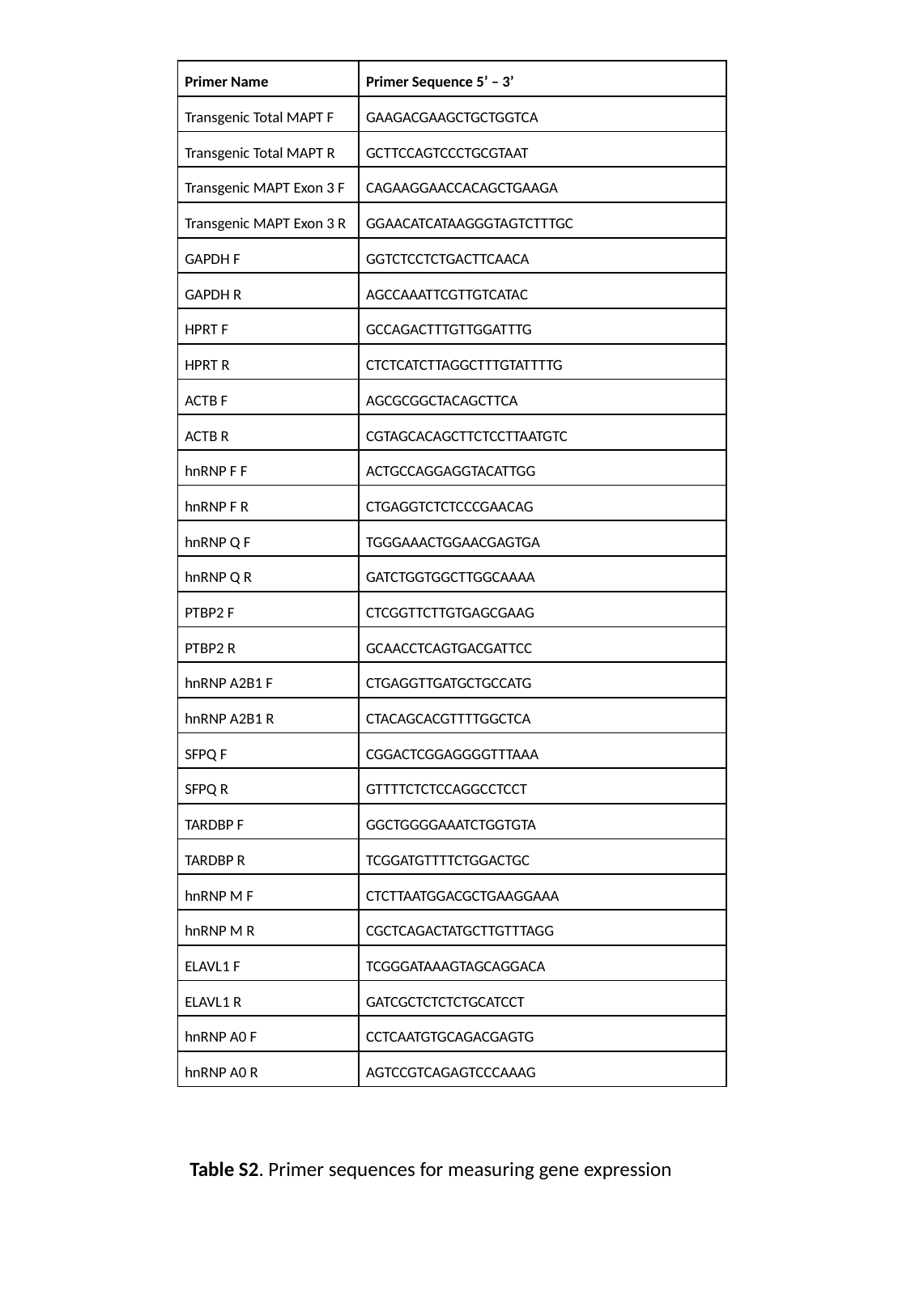

| Primer Name | Primer Sequence 5’ – 3’ |
| --- | --- |
| Transgenic Total MAPT F | GAAGACGAAGCTGCTGGTCA |
| Transgenic Total MAPT R | GCTTCCAGTCCCTGCGTAAT |
| Transgenic MAPT Exon 3 F | CAGAAGGAACCACAGCTGAAGA |
| Transgenic MAPT Exon 3 R | GGAACATCATAAGGGTAGTCTTTGC |
| GAPDH F | GGTCTCCTCTGACTTCAACA |
| GAPDH R | AGCCAAATTCGTTGTCATAC |
| HPRT F | GCCAGACTTTGTTGGATTTG |
| HPRT R | CTCTCATCTTAGGCTTTGTATTTTG |
| ACTB F | AGCGCGGCTACAGCTTCA |
| ACTB R | CGTAGCACAGCTTCTCCTTAATGTC |
| hnRNP F F | ACTGCCAGGAGGTACATTGG |
| hnRNP F R | CTGAGGTCTCTCCCGAACAG |
| hnRNP Q F | TGGGAAACTGGAACGAGTGA |
| hnRNP Q R | GATCTGGTGGCTTGGCAAAA |
| PTBP2 F | CTCGGTTCTTGTGAGCGAAG |
| PTBP2 R | GCAACCTCAGTGACGATTCC |
| hnRNP A2B1 F | CTGAGGTTGATGCTGCCATG |
| hnRNP A2B1 R | CTACAGCACGTTTTGGCTCA |
| SFPQ F | CGGACTCGGAGGGGTTTAAA |
| SFPQ R | GTTTTCTCTCCAGGCCTCCT |
| TARDBP F | GGCTGGGGAAATCTGGTGTA |
| TARDBP R | TCGGATGTTTTCTGGACTGC |
| hnRNP M F | CTCTTAATGGACGCTGAAGGAAA |
| hnRNP M R | CGCTCAGACTATGCTTGTTTAGG |
| ELAVL1 F | TCGGGATAAAGTAGCAGGACA |
| ELAVL1 R | GATCGCTCTCTCTGCATCCT |
| hnRNP A0 F | CCTCAATGTGCAGACGAGTG |
| hnRNP A0 R | AGTCCGTCAGAGTCCCAAAG |
Table S2. Primer sequences for measuring gene expression

## Slide 18
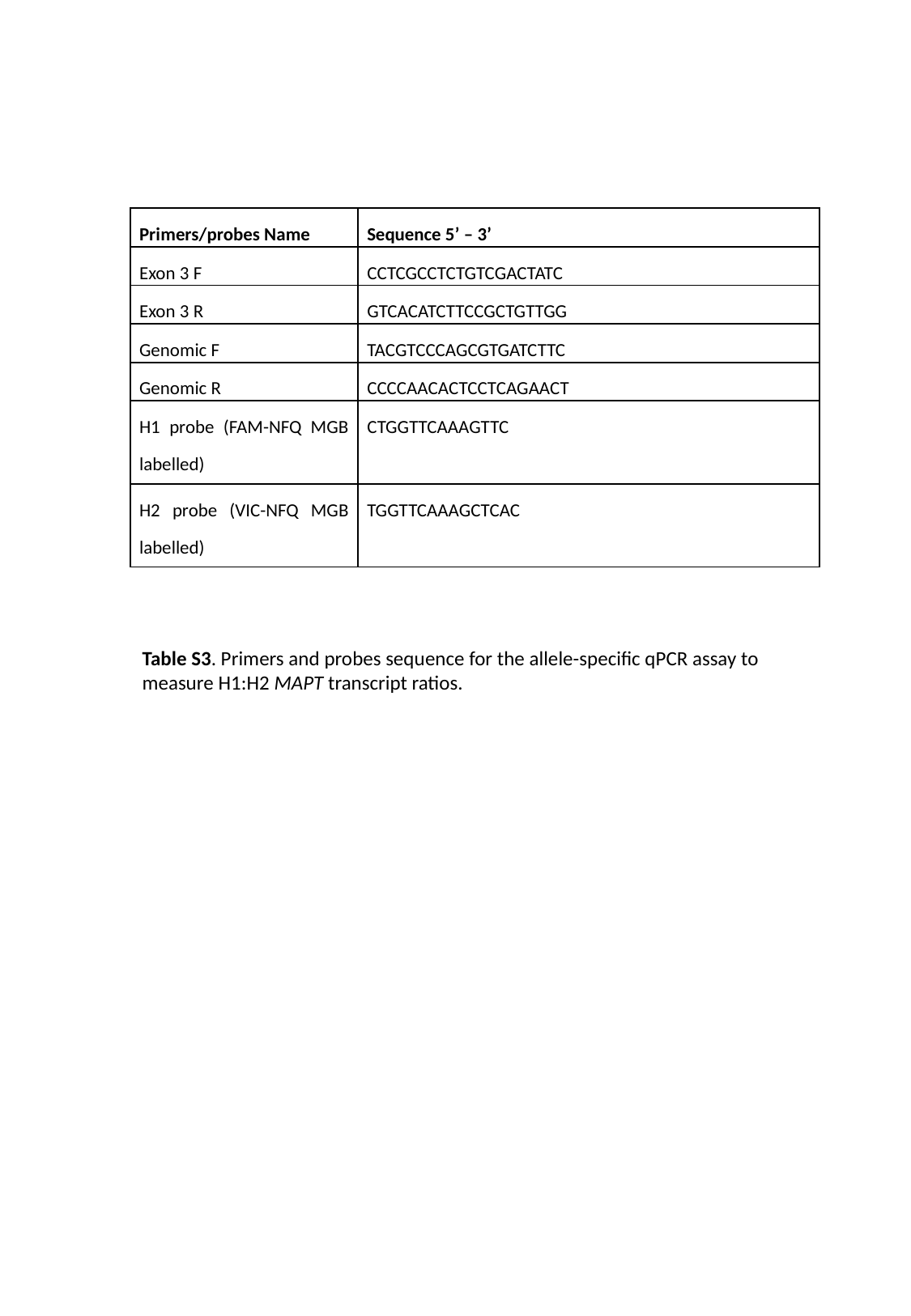

| Primers/probes Name | Sequence 5’ – 3’ |
| --- | --- |
| Exon 3 F | CCTCGCCTCTGTCGACTATC |
| Exon 3 R | GTCACATCTTCCGCTGTTGG |
| Genomic F | TACGTCCCAGCGTGATCTTC |
| Genomic R | CCCCAACACTCCTCAGAACT |
| H1 probe (FAM-NFQ MGB labelled) | CTGGTTCAAAGTTC |
| H2 probe (VIC-NFQ MGB labelled) | TGGTTCAAAGCTCAC |
Table S3. Primers and probes sequence for the allele-specific qPCR assay to measure H1:H2 MAPT transcript ratios.

## Slide 19
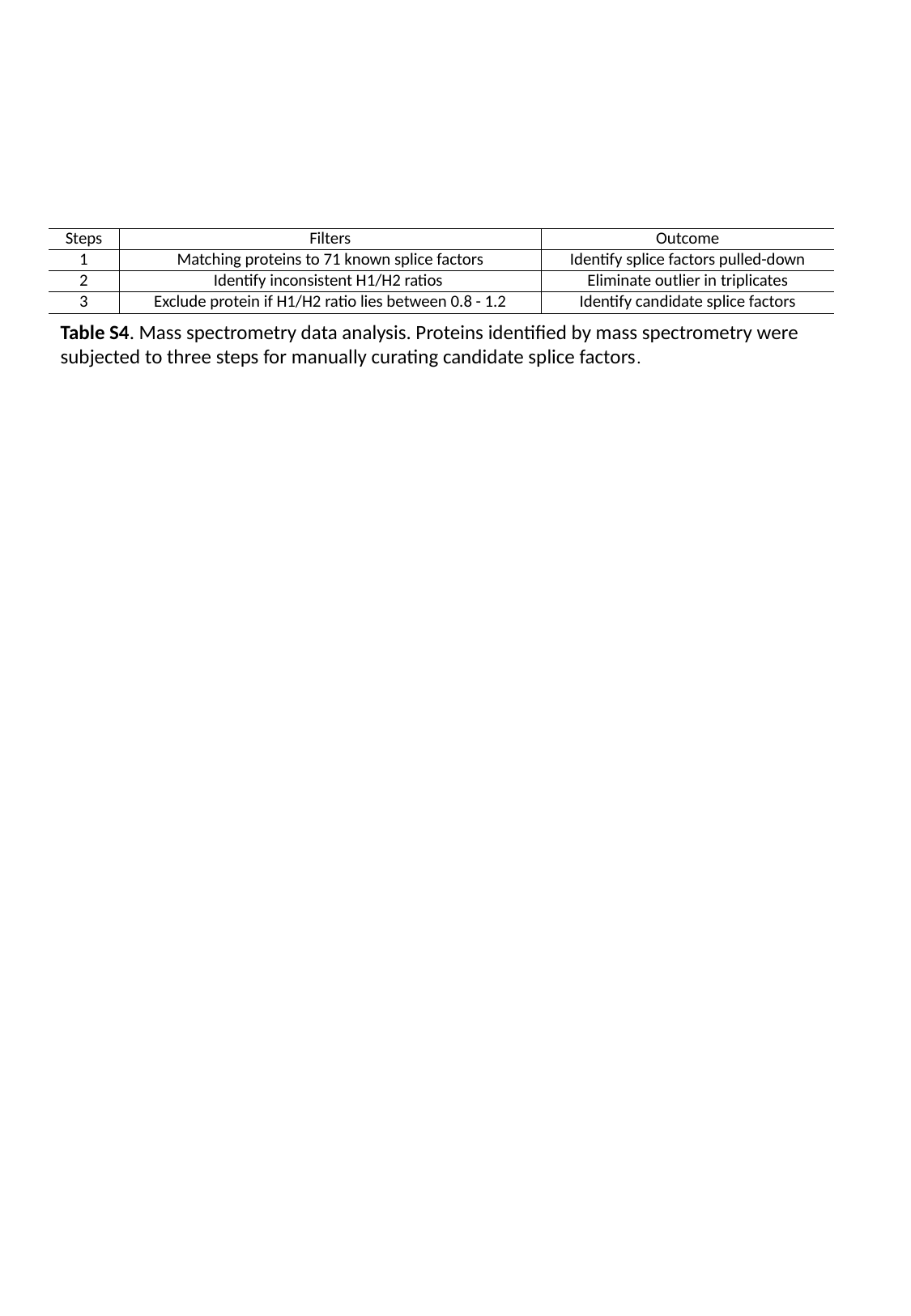

| Steps | Filters | Outcome |
| --- | --- | --- |
| 1 | Matching proteins to 71 known splice factors | Identify splice factors pulled-down |
| 2 | Identify inconsistent H1/H2 ratios | Eliminate outlier in triplicates |
| 3 | Exclude protein if H1/H2 ratio lies between 0.8 - 1.2 | Identify candidate splice factors |
Table S4. Mass spectrometry data analysis. Proteins identified by mass spectrometry were subjected to three steps for manually curating candidate splice factors.

## Slide 20
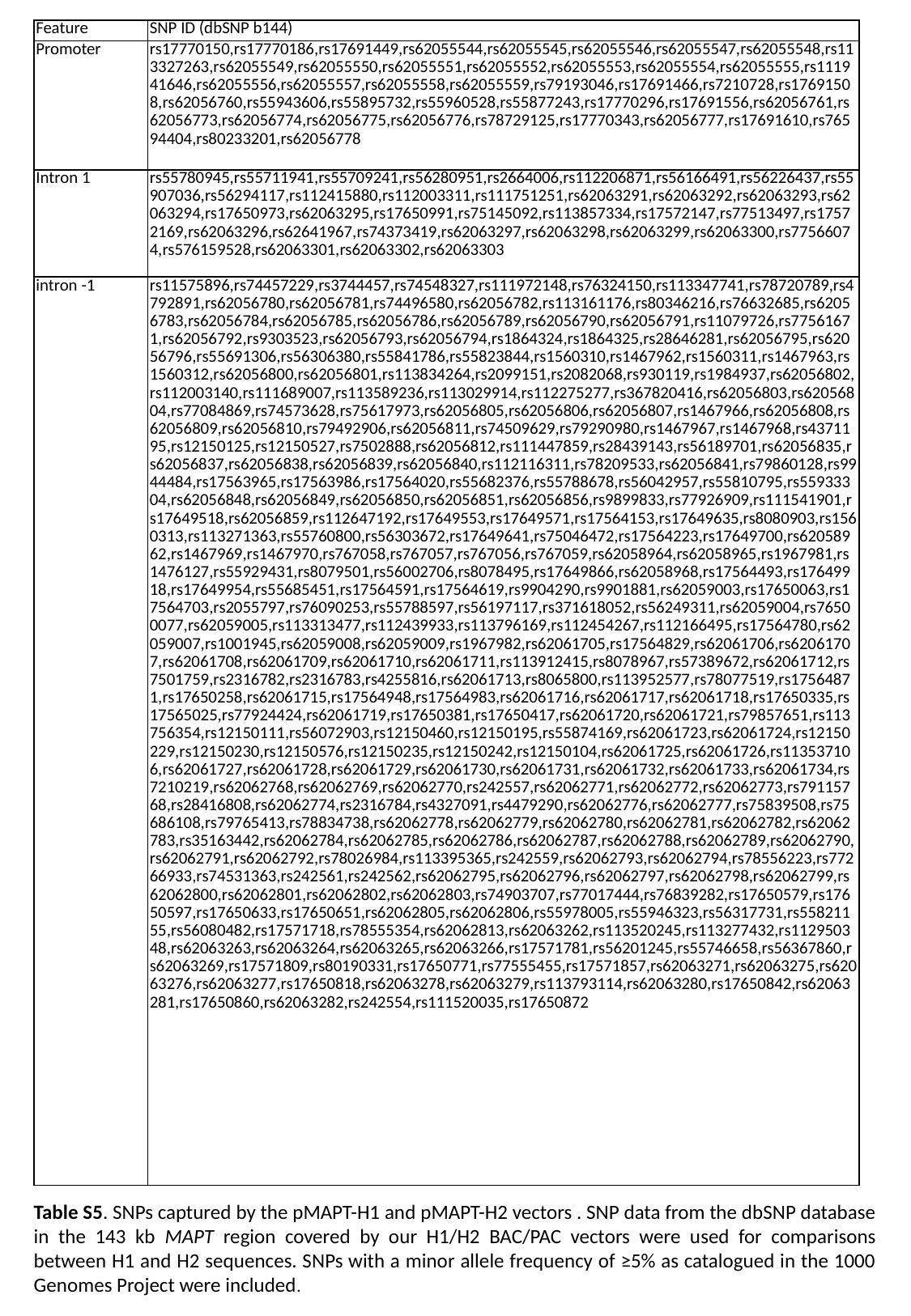

| Feature | SNP ID (dbSNP b144) |
| --- | --- |
| Promoter | rs17770150,rs17770186,rs17691449,rs62055544,rs62055545,rs62055546,rs62055547,rs62055548,rs113327263,rs62055549,rs62055550,rs62055551,rs62055552,rs62055553,rs62055554,rs62055555,rs111941646,rs62055556,rs62055557,rs62055558,rs62055559,rs79193046,rs17691466,rs7210728,rs17691508,rs62056760,rs55943606,rs55895732,rs55960528,rs55877243,rs17770296,rs17691556,rs62056761,rs62056773,rs62056774,rs62056775,rs62056776,rs78729125,rs17770343,rs62056777,rs17691610,rs76594404,rs80233201,rs62056778 |
| Intron 1 | rs55780945,rs55711941,rs55709241,rs56280951,rs2664006,rs112206871,rs56166491,rs56226437,rs55907036,rs56294117,rs112415880,rs112003311,rs111751251,rs62063291,rs62063292,rs62063293,rs62063294,rs17650973,rs62063295,rs17650991,rs75145092,rs113857334,rs17572147,rs77513497,rs17572169,rs62063296,rs62641967,rs74373419,rs62063297,rs62063298,rs62063299,rs62063300,rs77566074,rs576159528,rs62063301,rs62063302,rs62063303 |
| intron -1 | rs11575896,rs74457229,rs3744457,rs74548327,rs111972148,rs76324150,rs113347741,rs78720789,rs4792891,rs62056780,rs62056781,rs74496580,rs62056782,rs113161176,rs80346216,rs76632685,rs62056783,rs62056784,rs62056785,rs62056786,rs62056789,rs62056790,rs62056791,rs11079726,rs77561671,rs62056792,rs9303523,rs62056793,rs62056794,rs1864324,rs1864325,rs28646281,rs62056795,rs62056796,rs55691306,rs56306380,rs55841786,rs55823844,rs1560310,rs1467962,rs1560311,rs1467963,rs1560312,rs62056800,rs62056801,rs113834264,rs2099151,rs2082068,rs930119,rs1984937,rs62056802,rs112003140,rs111689007,rs113589236,rs113029914,rs112275277,rs367820416,rs62056803,rs62056804,rs77084869,rs74573628,rs75617973,rs62056805,rs62056806,rs62056807,rs1467966,rs62056808,rs62056809,rs62056810,rs79492906,rs62056811,rs74509629,rs79290980,rs1467967,rs1467968,rs4371195,rs12150125,rs12150527,rs7502888,rs62056812,rs111447859,rs28439143,rs56189701,rs62056835,rs62056837,rs62056838,rs62056839,rs62056840,rs112116311,rs78209533,rs62056841,rs79860128,rs9944484,rs17563965,rs17563986,rs17564020,rs55682376,rs55788678,rs56042957,rs55810795,rs55933304,rs62056848,rs62056849,rs62056850,rs62056851,rs62056856,rs9899833,rs77926909,rs111541901,rs17649518,rs62056859,rs112647192,rs17649553,rs17649571,rs17564153,rs17649635,rs8080903,rs1560313,rs113271363,rs55760800,rs56303672,rs17649641,rs75046472,rs17564223,rs17649700,rs62058962,rs1467969,rs1467970,rs767058,rs767057,rs767056,rs767059,rs62058964,rs62058965,rs1967981,rs1476127,rs55929431,rs8079501,rs56002706,rs8078495,rs17649866,rs62058968,rs17564493,rs17649918,rs17649954,rs55685451,rs17564591,rs17564619,rs9904290,rs9901881,rs62059003,rs17650063,rs17564703,rs2055797,rs76090253,rs55788597,rs56197117,rs371618052,rs56249311,rs62059004,rs76500077,rs62059005,rs113313477,rs112439933,rs113796169,rs112454267,rs112166495,rs17564780,rs62059007,rs1001945,rs62059008,rs62059009,rs1967982,rs62061705,rs17564829,rs62061706,rs62061707,rs62061708,rs62061709,rs62061710,rs62061711,rs113912415,rs8078967,rs57389672,rs62061712,rs7501759,rs2316782,rs2316783,rs4255816,rs62061713,rs8065800,rs113952577,rs78077519,rs17564871,rs17650258,rs62061715,rs17564948,rs17564983,rs62061716,rs62061717,rs62061718,rs17650335,rs17565025,rs77924424,rs62061719,rs17650381,rs17650417,rs62061720,rs62061721,rs79857651,rs113756354,rs12150111,rs56072903,rs12150460,rs12150195,rs55874169,rs62061723,rs62061724,rs12150229,rs12150230,rs12150576,rs12150235,rs12150242,rs12150104,rs62061725,rs62061726,rs113537106,rs62061727,rs62061728,rs62061729,rs62061730,rs62061731,rs62061732,rs62061733,rs62061734,rs7210219,rs62062768,rs62062769,rs62062770,rs242557,rs62062771,rs62062772,rs62062773,rs79115768,rs28416808,rs62062774,rs2316784,rs4327091,rs4479290,rs62062776,rs62062777,rs75839508,rs75686108,rs79765413,rs78834738,rs62062778,rs62062779,rs62062780,rs62062781,rs62062782,rs62062783,rs35163442,rs62062784,rs62062785,rs62062786,rs62062787,rs62062788,rs62062789,rs62062790,rs62062791,rs62062792,rs78026984,rs113395365,rs242559,rs62062793,rs62062794,rs78556223,rs77266933,rs74531363,rs242561,rs242562,rs62062795,rs62062796,rs62062797,rs62062798,rs62062799,rs62062800,rs62062801,rs62062802,rs62062803,rs74903707,rs77017444,rs76839282,rs17650579,rs17650597,rs17650633,rs17650651,rs62062805,rs62062806,rs55978005,rs55946323,rs56317731,rs55821155,rs56080482,rs17571718,rs78555354,rs62062813,rs62063262,rs113520245,rs113277432,rs112950348,rs62063263,rs62063264,rs62063265,rs62063266,rs17571781,rs56201245,rs55746658,rs56367860,rs62063269,rs17571809,rs80190331,rs17650771,rs77555455,rs17571857,rs62063271,rs62063275,rs62063276,rs62063277,rs17650818,rs62063278,rs62063279,rs113793114,rs62063280,rs17650842,rs62063281,rs17650860,rs62063282,rs242554,rs111520035,rs17650872 |
Table S5. SNPs captured by the pMAPT-H1 and pMAPT-H2 vectors . SNP data from the dbSNP database in the 143 kb MAPT region covered by our H1/H2 BAC/PAC vectors were used for comparisons between H1 and H2 sequences. SNPs with a minor allele frequency of ≥5% as catalogued in the 1000 Genomes Project were included.

## Slide 21
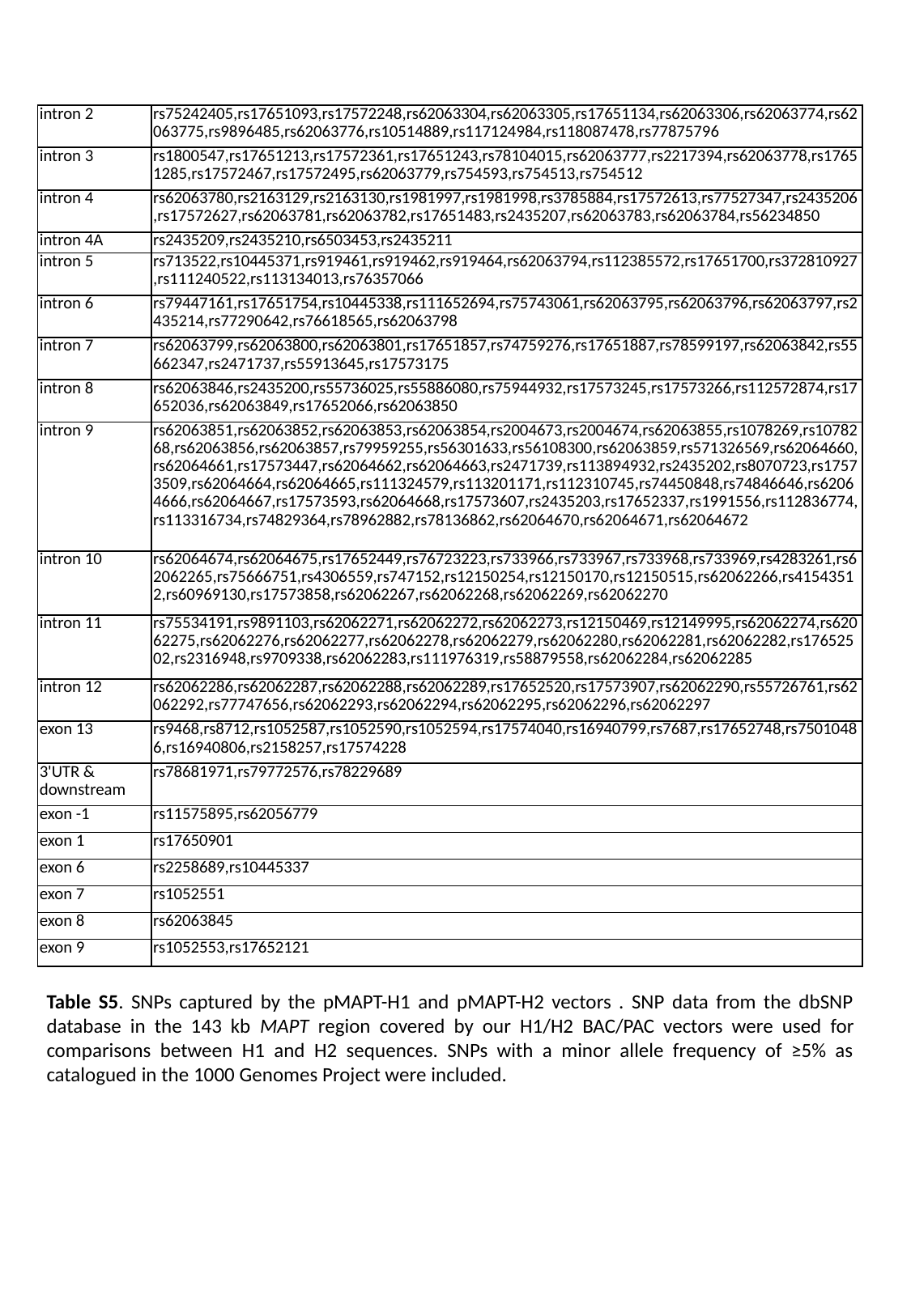

| intron 2 | rs75242405,rs17651093,rs17572248,rs62063304,rs62063305,rs17651134,rs62063306,rs62063774,rs62063775,rs9896485,rs62063776,rs10514889,rs117124984,rs118087478,rs77875796 |
| --- | --- |
| intron 3 | rs1800547,rs17651213,rs17572361,rs17651243,rs78104015,rs62063777,rs2217394,rs62063778,rs17651285,rs17572467,rs17572495,rs62063779,rs754593,rs754513,rs754512 |
| intron 4 | rs62063780,rs2163129,rs2163130,rs1981997,rs1981998,rs3785884,rs17572613,rs77527347,rs2435206,rs17572627,rs62063781,rs62063782,rs17651483,rs2435207,rs62063783,rs62063784,rs56234850 |
| intron 4A | rs2435209,rs2435210,rs6503453,rs2435211 |
| intron 5 | rs713522,rs10445371,rs919461,rs919462,rs919464,rs62063794,rs112385572,rs17651700,rs372810927,rs111240522,rs113134013,rs76357066 |
| intron 6 | rs79447161,rs17651754,rs10445338,rs111652694,rs75743061,rs62063795,rs62063796,rs62063797,rs2435214,rs77290642,rs76618565,rs62063798 |
| intron 7 | rs62063799,rs62063800,rs62063801,rs17651857,rs74759276,rs17651887,rs78599197,rs62063842,rs55662347,rs2471737,rs55913645,rs17573175 |
| intron 8 | rs62063846,rs2435200,rs55736025,rs55886080,rs75944932,rs17573245,rs17573266,rs112572874,rs17652036,rs62063849,rs17652066,rs62063850 |
| intron 9 | rs62063851,rs62063852,rs62063853,rs62063854,rs2004673,rs2004674,rs62063855,rs1078269,rs1078268,rs62063856,rs62063857,rs79959255,rs56301633,rs56108300,rs62063859,rs571326569,rs62064660,rs62064661,rs17573447,rs62064662,rs62064663,rs2471739,rs113894932,rs2435202,rs8070723,rs17573509,rs62064664,rs62064665,rs111324579,rs113201171,rs112310745,rs74450848,rs74846646,rs62064666,rs62064667,rs17573593,rs62064668,rs17573607,rs2435203,rs17652337,rs1991556,rs112836774,rs113316734,rs74829364,rs78962882,rs78136862,rs62064670,rs62064671,rs62064672 |
| intron 10 | rs62064674,rs62064675,rs17652449,rs76723223,rs733966,rs733967,rs733968,rs733969,rs4283261,rs62062265,rs75666751,rs4306559,rs747152,rs12150254,rs12150170,rs12150515,rs62062266,rs41543512,rs60969130,rs17573858,rs62062267,rs62062268,rs62062269,rs62062270 |
| intron 11 | rs75534191,rs9891103,rs62062271,rs62062272,rs62062273,rs12150469,rs12149995,rs62062274,rs62062275,rs62062276,rs62062277,rs62062278,rs62062279,rs62062280,rs62062281,rs62062282,rs17652502,rs2316948,rs9709338,rs62062283,rs111976319,rs58879558,rs62062284,rs62062285 |
| intron 12 | rs62062286,rs62062287,rs62062288,rs62062289,rs17652520,rs17573907,rs62062290,rs55726761,rs62062292,rs77747656,rs62062293,rs62062294,rs62062295,rs62062296,rs62062297 |
| exon 13 | rs9468,rs8712,rs1052587,rs1052590,rs1052594,rs17574040,rs16940799,rs7687,rs17652748,rs75010486,rs16940806,rs2158257,rs17574228 |
| 3'UTR & downstream | rs78681971,rs79772576,rs78229689 |
| exon -1 | rs11575895,rs62056779 |
| exon 1 | rs17650901 |
| exon 6 | rs2258689,rs10445337 |
| exon 7 | rs1052551 |
| exon 8 | rs62063845 |
| exon 9 | rs1052553,rs17652121 |
Table S5. SNPs captured by the pMAPT-H1 and pMAPT-H2 vectors . SNP data from the dbSNP database in the 143 kb MAPT region covered by our H1/H2 BAC/PAC vectors were used for comparisons between H1 and H2 sequences. SNPs with a minor allele frequency of ≥5% as catalogued in the 1000 Genomes Project were included.

## Slide 22
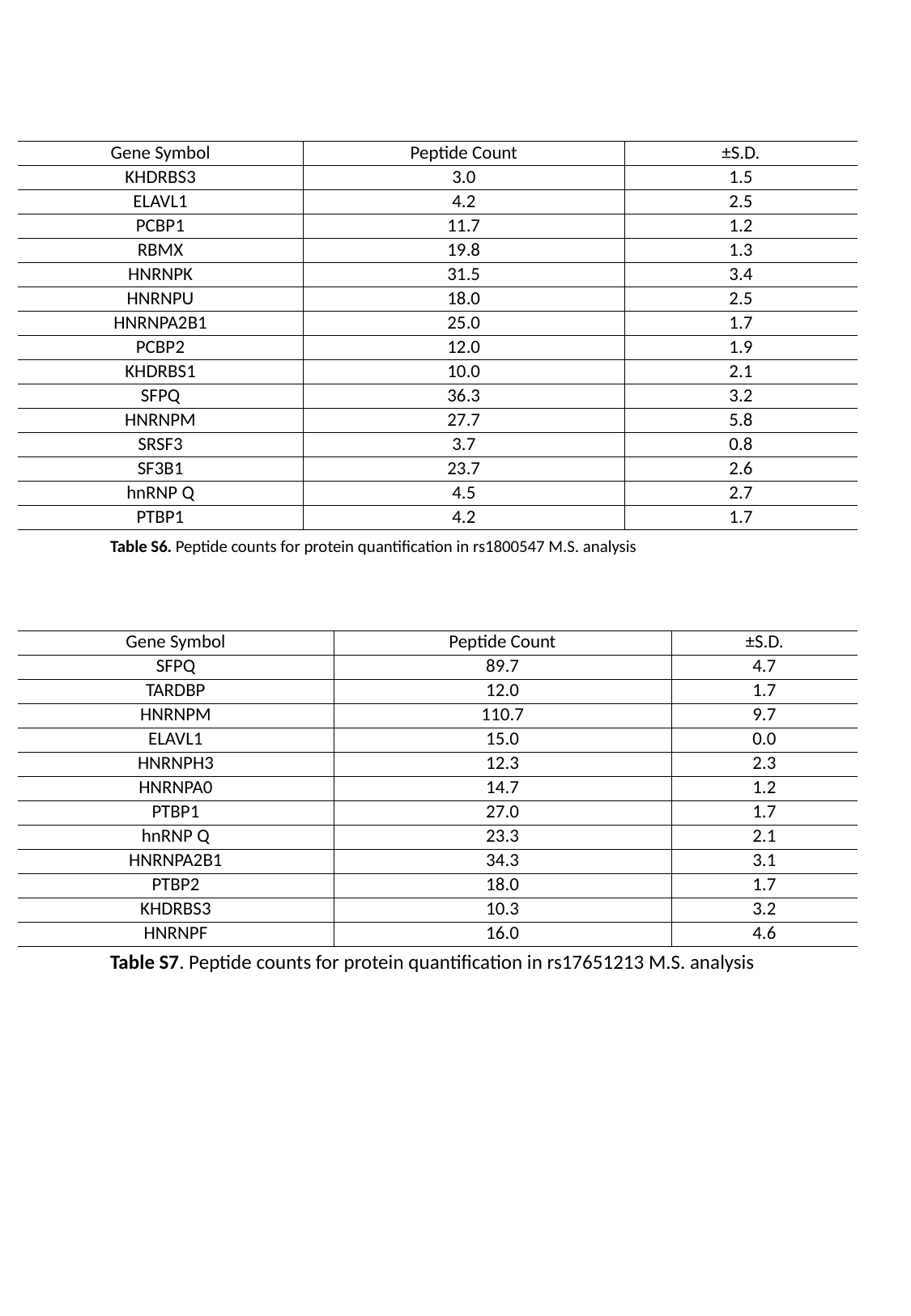

| | | |
| --- | --- | --- |
| Gene Symbol | Peptide Count | ±S.D. |
| KHDRBS3 | 3.0 | 1.5 |
| ELAVL1 | 4.2 | 2.5 |
| PCBP1 | 11.7 | 1.2 |
| RBMX | 19.8 | 1.3 |
| HNRNPK | 31.5 | 3.4 |
| HNRNPU | 18.0 | 2.5 |
| HNRNPA2B1 | 25.0 | 1.7 |
| PCBP2 | 12.0 | 1.9 |
| KHDRBS1 | 10.0 | 2.1 |
| SFPQ | 36.3 | 3.2 |
| HNRNPM | 27.7 | 5.8 |
| SRSF3 | 3.7 | 0.8 |
| SF3B1 | 23.7 | 2.6 |
| hnRNP Q | 4.5 | 2.7 |
| PTBP1 | 4.2 | 1.7 |
Table S6. Peptide counts for protein quantification in rs1800547 M.S. analysis
| | | |
| --- | --- | --- |
| Gene Symbol | Peptide Count | ±S.D. |
| SFPQ | 89.7 | 4.7 |
| TARDBP | 12.0 | 1.7 |
| HNRNPM | 110.7 | 9.7 |
| ELAVL1 | 15.0 | 0.0 |
| HNRNPH3 | 12.3 | 2.3 |
| HNRNPA0 | 14.7 | 1.2 |
| PTBP1 | 27.0 | 1.7 |
| hnRNP Q | 23.3 | 2.1 |
| HNRNPA2B1 | 34.3 | 3.1 |
| PTBP2 | 18.0 | 1.7 |
| KHDRBS3 | 10.3 | 3.2 |
| HNRNPF | 16.0 | 4.6 |
Table S7. Peptide counts for protein quantification in rs17651213 M.S. analysis
